# Comprehensive landscape of the renin-angiotensin system in Pan-cancer: a potential downstream mediated mechanism of SARS-CoV-2

**DOI:** 10.7150/ijbs.53312

**Published:** 2021-09-03

**Authors:** Yuqing Cui, Fengzhi Chen, Jiayi Gao, Mengxia Lei, Dandan Wang, Xiaoying Jin, Yan Guo, Liying Shan, Xuesong Chen

**Affiliations:** The Fourth Department of Medical Oncology, Harbin Medical University Cancer Hospital, Harbin 150040, China.

**Keywords:** SARS-CoV-2, RAS family, Pan-cancer, Immune infiltration, ACE2

## Abstract

**Background:** SARS-CoV-2, the cause of the worldwide COVID-19 pandemic, utilizes the mechanism of binding to ACE2 (a crucial component of the renin-angiotensin system [RAS]), subsequently mediating a secondary imbalance of the RAS family and leading to severe injury to the host. However, very few studies have been conducted to reveal the mechanism behind the effect of SARS-CoV-2 on tumors.

**Methods:** Demographic data extracted from 33 cancer types and over 10,000 samples were employed to determine the comprehensive landscape of the RAS. Expression distribution, pretranscriptional and posttranscriptional regulation and posttranslational modifications (PTMs) as well as genomic alterations, DNA methylation and m6A modification were analyzed in both tissue and cell lines. The clinical phenotype, prognostic value and significance of the RAS during immune infiltration were identified.

**Results:** Low expression of AGTR1 was common in tumors compared to normal tissues, while very low expression of AGTR2 and MAS1 was detected in both tissues and cell lines. Differential expression patterns of ACE in ovarian serous cystadenocarcinoma (OV) and kidney renal clear cell carcinoma (KIRC) were correlated with ubiquitin modification involving E3 ligases. Genomic alterations of the RAS family were infrequent across TCGA pan-cancer program, and ACE had the highest alteration frequency compared with other members. Low expression of AGTR1 may result from hypermethylation in the promoter. Downregulation of RAS family was linked to higher clinical stage and worse survival (as measured by disease-specific survival [DSS], overall survival [OS] or progression-free interval [PFI]), especially for ACE2 and AGTR1 in KIRC. ACE-AGTR1, a classical axis of the RAS family related to immune infiltration, was positively correlated with M2-type macrophages, cancer-associated fibroblasts (CAFs) and immune checkpoint genes in most cancers.

**Conclusion:** ACE, ACE2, AGT and AGTR1 were differentially expressed in 33 types of cancers. PTM of RAS family was found to rely on ubiquitination. ACE2 and AGTR1 might serve as independent prognostic factors for LGG and KIRC. SARS-CoV-2 might modify the tumor microenvironment by regulating the RAS family, thus affecting the biological processes of cancer.

## Introduction

The COVID-19 outbreak caused by SARS-CoV-2 infection started in December 2019 and has become a worldwide emergency, posing a severe threat to global health. SARS-CoV-2, a typical RNA virus similar to SARS-CoV and MERS-CoV, belongs to the coronaviridae family 1. SARS-CoV-2 is similar to SARS-CoV in its ability to interact with angiotensin-converting enzyme 2 (ACE2) in host cell membranes via spiny dendritic proteins (S proteins) 2, 3. The binding affinity of SARS-CoV-2 for ACE2 is 10-20-fold higher than that of SARS-CoV, which contributes to its highly infectious nature and severe damage to target organs 4. By binding to the ACE2 receptor on the host cell surface, SARS-CoV-2 induces a decrease in ACE2 expression, leading to the accumulation of angiotensin II (Ang II) 5. Evidence has proven that Ang II can induce the release of inflammatory factors and produce subsequent damage to target organs 6, 7. Although previous studies have pointed out that SARS-CoV-2 infection might be related to higher susceptibility to and worse prognosis in cancer, specific biological mechanisms have not been elucidated 8-10. In addition, the potential roles of the SARS-CoV-2 receptor ACE2 and the renin-angiotensin system (RAS) in cancer patients with SARS-CoV-2 infection remain unclear.

**Figure 1:**
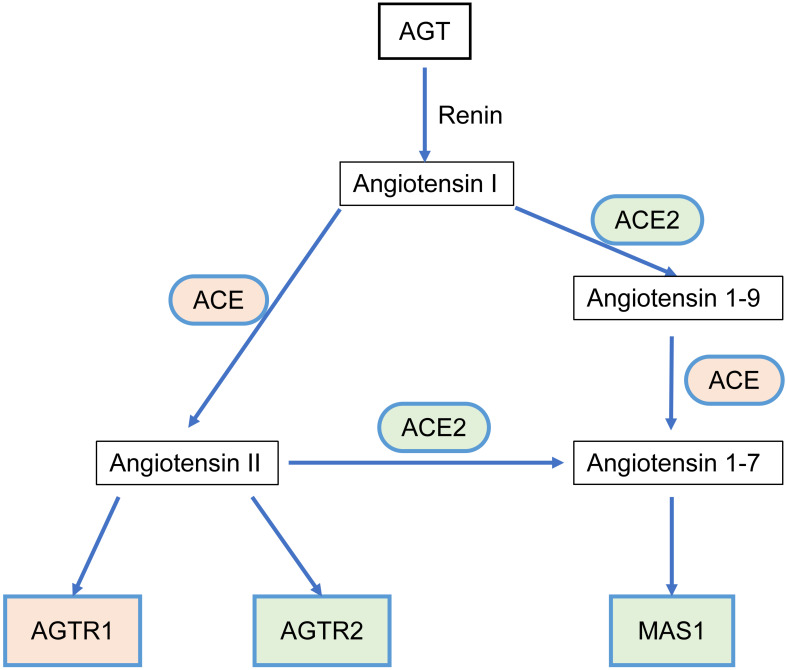
The relationship scheme of RAS family. AGT could be converted to Angiotensin I (Ang I) by renin. Ang I is converted to Ang II or Ang (1-9) by ACE or ACE2, respectively. Ang II is converted to Ang (1-7) by ACE2. Ang II could bind to two types of seven-transmembrane G protein-coupled receptors: AGTR1 and AGTR2. Ang (1-7) could bind to MAS1.

The RAS acts as a classic endocrine regulatory machinery involved in numerous physiological/pathological regulatory processes 11. Angiotensinogen (AGT), a precursor of the angiotensin peptide, is converted to angiotensin I (Ang I) by renin 12. Then, Ang I is converted to Ang II or Ang (1-9) by angiotensin-converting enzyme (ACE) or ACE2, respectively. Ang II is converted to Ang (1-7) by ACE2. Ang II can bind to two types of seven-transmembrane G protein-coupled receptors: angiotensin II type I receptor (AGTR1) and angiotensin II type II receptor (AGTR2). On the other hand, Ang (1-7) binds to the Mas1 receptor (MAS1). Such a system constitutes two significant axes: the classical ACE/Ang II/AGTR1 branch and the counter-regulatory ACE2/Ang (1-7)/MAS1 branch (**Figure 1**). Dynamic homeostasis of the RAS is maintained throughout the lifetime. When imbalance occurs, pathophysiological processes are triggered. In addition to the systemic RAS, local RASs also exist in the lungs, brain, kidneys, and tumors and are dysregulated during pathological processes. Several studies in breast, lung, and colon cancer have proposed that the classical axis (ACE/Ang II/AGTR1) can activate multiple downstream signaling pathways (PI3K-AKT, MAPK, and TGF-β) and biological factors to promote tumor cell proliferation, invasion, and anti-apoptosis mechanisms 13, 14. AGTR2 plays a physiological role opposite that of AGTR1 15-18. The alternative regulatory axis (ACE2/Ang 1-7/MAS1) has anti-tumor effects 19, 20.

The SARS-CoV-2 receptor ACE2 and the RAS family are involved in the development and progression of multiple solid tumors 21-23. However, no studies with large sample sizes using high-throughput multiomics analyses have been performed. In this study, we comprehensively integrated and analyzed the molecular characteristics of 6 significant members of the RAS family across pan-cancer: AGT, ACE, ACE2, AGTR1, AGTR2, and MAS1. Based on a dataset comprising 33 types of cancer and over 10,000 samples, a multiomics landscape of the RAS family was determined to assess expression distribution, transcription, posttranscriptional regulation, translation, posttranslational modification (PTM), genomic alterations, epigenetic modifications, clinical features, prognostic utility, functional prediction, and immune infiltration. The study presents multifaceted biological impacts of the RAS family and provides clues and directions for future research on the mode of action of SARS-CoV-2 in cancer.

## Materials and Methods

### Expression of RAS family

The RNA-seq data of GTEx 24 and TCGA and clinical information were extracted from UCSC Xena 25. The Wilcoxon test was used to calculate the differential expression between normal and tumor tissues. An absolute value of log2 fold change (logFC) greater than one with a significant P-value less than 0.01 indicated differential expression. The normalized proteomics data of ACE, ACE2, and AGT in 7 cancer types (breast invasive carcinoma [BRCA], colon adenocarcinoma [COAD], glioblastoma [GBM], kidney renal clear cell carcinoma [KIRC], lung adenocarcinoma [LUAD], ovarian serous cystadenocarcinoma [OV], and uterine corpus endometrial carcinoma [UCEC]) acquired from CPTAC Data Portal 26 were used to compare expression between tumor and normal tissues in a two-group test. RNA-seq data from the Broad Institute CCLE project 27 and proteomics data from DepMap (https://depmap.org/portal/) were utilized to compare mRNA and protein expression, respectively, in human cancer cell lines. A Cox proportional hazards model was employed for multivariate assessment of genes with significant prognostic value in the univariate analysis. The survival information for the GEO analysis of RAS family was acquired from PrognoScan 28.

### PTM analysis of RAS family

The predicted ubiquitin ligases (E3) related to the RAS were discovered via UbiBrowser 29. The database contains previously reported E3-substrate interactions and predicted E3-substrate interactions. The associations between target genes and E3 ligases were calculated by Pearson's coefficient analysis across 33 types of cancers in TCGA.

### Transcriptional and posttranscriptional analyses of RAS family

Transcription factors (TFs) and microRNAs (miRNAs) targeting each RAS member were discovered via RegNetwork 30. LncRNA2Target2.0 31 was used to extract lncRNA-RAS member associations. The RNA-seq data of miRNAs were downloaded from UCSC Xena. The correlation between TFs or miRNAs and RAS members was calculated by Pearson's coefficient analysis.

### Genomic alteration of RAS family

Genetic alterations of RAS family identified in TCGA pancancer and CCLE data were analyzed with cBioportal 32. The fusion genes affecting RAS family were identified with TCGA Fusion Gene Database 33, an atlas comprised of 20,731 fusion transcripts identified through analysis of RNA-seq data from 33 cancer types and analyzed using the PRADA pipeline 34. The higher the E-value was, the lower the similarity shared between the two genes. A tier system was used to rank the fidelity of fusion transcripts. From tier 1 to tier 4, fidelity decreased.

### Methylation analysis of RAS family

The differential DNA methylation of RAS family between tumor and adjacent normal tissues, the correlation between DNA methylation and gene expression, and the predictive value of hypermethylation of RAS family for prognosis were assessed via GSCALite 35. M6A modification of RAS family was assessed with m6A2Target 36, which includes data regarding target genes of writers, erasers, and readers (WERs) involved in m6A modification derived from both low-throughput experiments and high-throughput sequencing.

### Enrichment analysis of RAS family

Protein-protein interactions (PPIs) of RAS family were predicted by GeneMANIA 37 and displayed in a network structured by Cytoscape 3.7.2. The RAS family and interacting proteins were subjected to GO and KEGG enrichment analyses in Metascape 38. The RNA-seq data of calu-3 and A549 cells extracted from GSE147507 39 were normalized by DESeq2 to conduct GSEA. GSEA was performed using the R 3.6.2 'GSEABase' and 'clusterprofiler' package, and only gene sets with a P-value<0.05 and Q-value<0.25 were considered significant. Raw read counts of calu-3 and A549 cells extracted from GSE147507 were processed GSVA using Poisson parameters. GSVA was performed using the R 3.6.2 'GSVA' package; only gene sets with a P-value<0.05 were considered significant.

### Immune analysis

The R package 'estimate' was used to evaluate the ratio of immune-stromal components in TCGA pan-cancer data, and three kinds of scores were determined: ImmuneScore, StromalScore, and ESTIMATEScore. The higher the respective score was, the larger the ratio of the corresponding components in each type of cancer. The correlation of tumor-infiltrating cells (TICs) with RAS family expression was assessed in various databases, including CIBERSORT, TIMER, xCell, EPIC, MCP-counter, and QUANTISIQ, with TIMER2.0 40. Somatic mutation data derived from the VarScan2 Variant Aggregation and Masking workflow were downloaded from UCSC Xena to assess the tumor mutational burden (TMB) in TCGA pan-cancer datasets. Information on microsatellite instability (MSI) of each sample in 33 cancer datasets from TCGA was downloaded from the FireBrowse database (http://www.firebrowse.org/).

## Results

### Expression profiling of RAS family in pan-cancer

**Figure 2:**
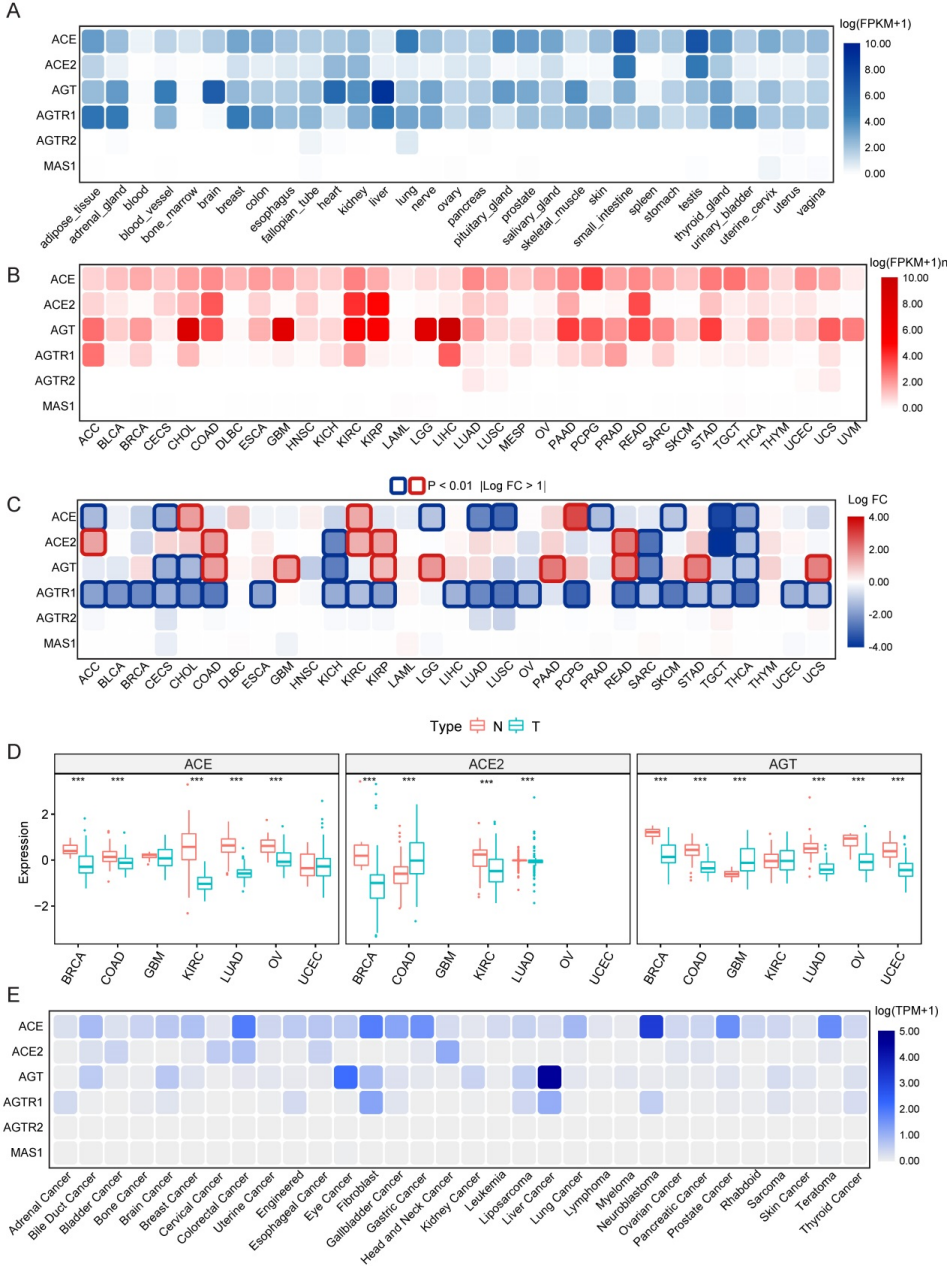
** Expression atlas of RAS family. (A)** Heatmap for relative mRNA expression of RAS in multiple normal tissues from GTEx. **(B)** Heatmap for mRNA expression of RAS in diverse tumor from TCGA 33 cancers. **(C)** Differential expression pattern of RAS between tumor and normal tissues based on conjoint analyses of TCGA and GTEx. Upregulation in cancer tissue labeled in red; downregulation in cancer tissue labeled in blue; borders for statistically significant (P < 0.01, |logFC| > 1). **(D)** Differential protein expression of ACE, ACE2 and AGT between tumor and normal tissue in 7 cancers (BRCA, COAD, GBM, KIRC, LUAD, OV) from CPTAC. Blue for T indicates tumor tissues whereas red for N indicates normal tissues. **(E)** Heatmap for mRNA expression of RAS across cancer cell lines from DapMap database. *P < 0.05, **P < 0.01, ***P < 0.001.

Data on the mRNA expression and distribution of RAS family expression in 31 different normal human tissues were extracted from GTEx (**Figure 2A**). Prominent variant patterns of RAS family across distinct tissues appeared, with broad expression of ACE, ACE2, AGT, and AGTR1 and sparse expression of AGTR2 and MAS1. The highest expression of ACE and ACE2 was detected in both the small intestine and testis. Relatively high expression of ACE was discovered in the lungs. AGT was prominently expressed in the organ in which it is produced, the liver 41, and AGTR1 was highly expressed in adipose, breast, and liver tissue and the adrenal gland.

The mRNA expression distribution of RAS family across 33 TCGA cancers was assessed (**Figure 2B**). In tumor tissue, AGTR2 and MAS1 were weakly expressed; in contrast, ACE and AGT presented broad expression across 33 cancer types. Notably, ACE2 was abundantly expressed in KIRC, kidney renal papillary cell carcinoma (KIRP), COAD and rectum adenocarcinoma (READ), indicating that these four cancers may be more vulnerable than others when exposed to SARS-CoV-2. AGT showed maximal expression in multiple cancers, including liver hepatocellular carcinoma (LIHC), cholangiocarcinoma (CHOL), GBM, low-grade glioma (LGG), KIRC and KIRP. Differential expression of RAS family across 31 types of cancers (**Figure 2C**) was profiled through combined analysis of TCGA and GTEx data. With significance cutoffs of |log FC| >1 and P-value < 0.01, AGTR1 exhibited widespread low expression in pan-cancer. ACE was only highly expressed in PCPG, CHOL, and KIRC tumor tissues, whereas it was expressed at low levels in 7 types of cancers. Interestingly, ACE2 and AGT were substantially expressed in tumor tissues compared to normal counterparts in cancers with high expression, such as KIRP, COAD and READ. The same trend was observed in the low expression groups in KICH, sarcoma (SARC) and thyroid carcinoma (THCA).

Because proteins and not mRNAs ultimately exert biological functions, the protein levels of ACE, ACE2, and AGT were explored in BRCA, COAD, GBM, KIRC, LUAD, OV and UCEC using the CPTAC database (**Figure 2D**). Interestingly, ACE protein levels were lower in KIRC and OV than in normal tissues. The expression of ACE2 in both KIRC and LUAD was lower than that in the corresponding normal tissues. Similarly, lower expression of AGT in tumor tissues than normal tissues was observed in BRCA, COAD, and LUAD. These results indicate the existence of additional modulatory mechanisms (posttranscriptional processes) affecting ACE, ACE2, and AGT.

Given the changes in tumor tissues over time and the spatial heterogeneity of tumor tissues as well as their complex microenvironments, cancer cell lines can reflect the expression abundance of genes more precisely than tumor tissues. Here, we utilized RNA-seq data of cell lines representing 32 cancer types from CCLE to identify the distribution of mRNA expression (**Figure 2E**) and protein levels (**Supplementary Figure 1**) of RAS family. Notably, AGTR2 and MAS1 were minimally expressed in both tissues and cell lines. Consistent with its expression in tissues, AGT was maximally expressed in liver cancer and relatively highly expressed in bile duct carcinoma, kidney cancer and brain cancer, partially corresponding to the results in LGG and GBM tissues. The AGTR1 abundance in liver cancer and adrenal cancer may provide a clue for tumor origin. Nevertheless, a discordant expression trend of ACE and ACE2 between cell lines and tissues was discovered, likely stemming from the relatively small sample size and complexity in tissues.

### PTM of RAS family in pan-cancer

**Figure 3:**
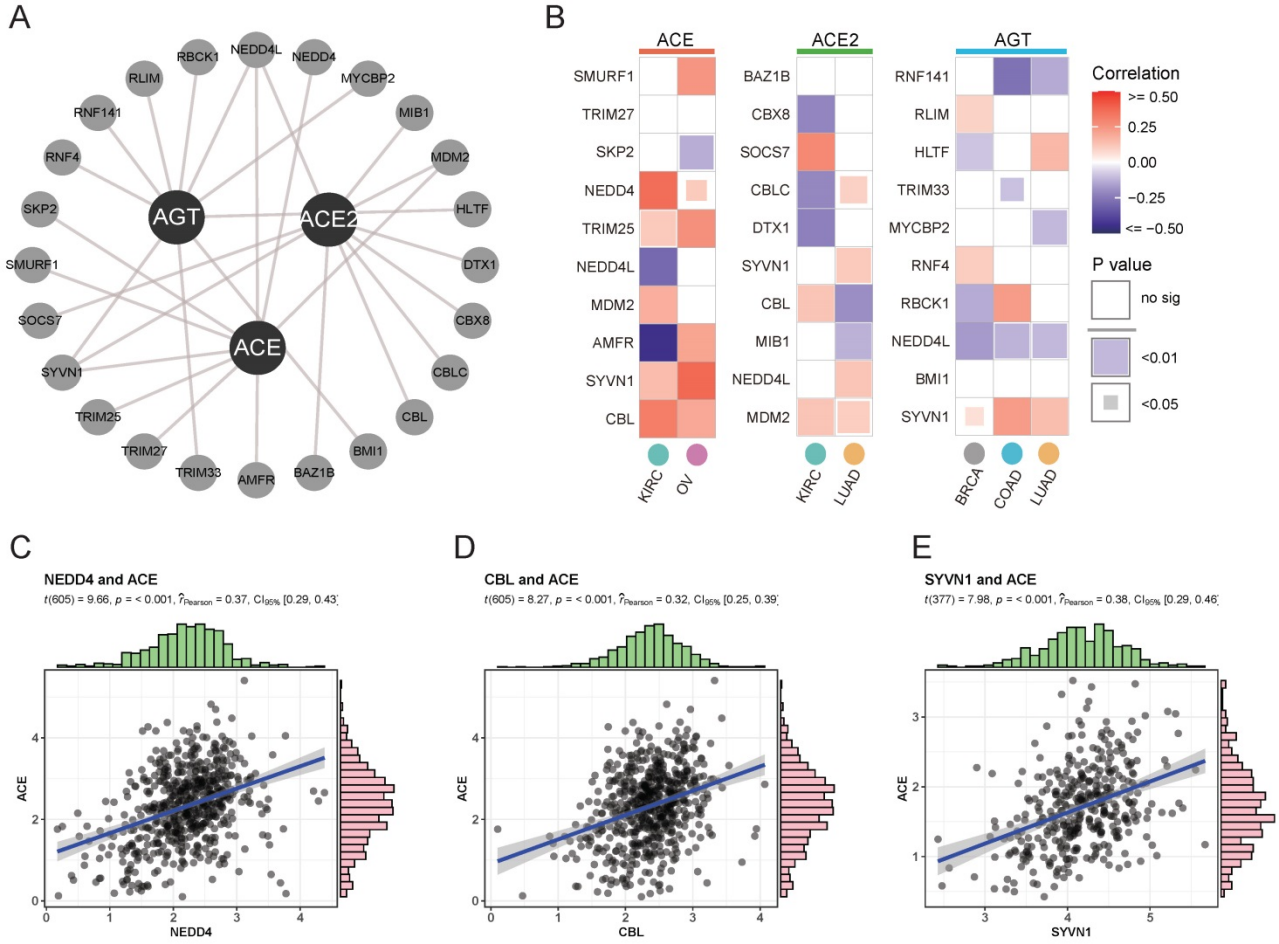
** Ubiquitylation analysis of RAS in specific cancers. (A)** Interaction network of ubiquitin ligase (E3 ligase) substrate and ACE, ACE2 and AGT from UbiBrowser. **(B)** Heatmap for correlation between E3 ligase and ACE, ACE2 and AGT in specific cancers, evaluated by Pearson's Coefficient. Red for positive correlation, whereas blue for negative. **(C)** Scatter plot for correlation between NEDD4 and ACE. **(D)** Scatter plot for correlation between CBL and ACE. **(E)** Scatter plot for correlation between SYVN1 and ACE.

To assess differential mRNA and protein expression, ubiquitination modifications affecting protein stability were explored as posttranslational factors. An interactive network of the top 10 significant E3 ligases (enzymes binding directly to target proteins during ubiquitination) affecting RAS family members was constructed (**Supplementary Figure 2** and **Figure 3A**). Variant expression of ACE, ACE2, and AGT was correlated with corresponding E3 ligase expression in cancer (**Figure 3B**). ACE was likely to bind NEDD4 (**Figure 3C**, Cor = 0.37) or CBL (**Figure 3D**, Cor = 0.32) in KIRC and SYVN1 (**Figure 3E**, Cor = 0.38) in OV. The results indicated that ubiquitination could take part in the protein degradation of ACE.

### Transcriptional and posttranscriptional modifications of RAS family in pan-cancer

**Figure 4:**
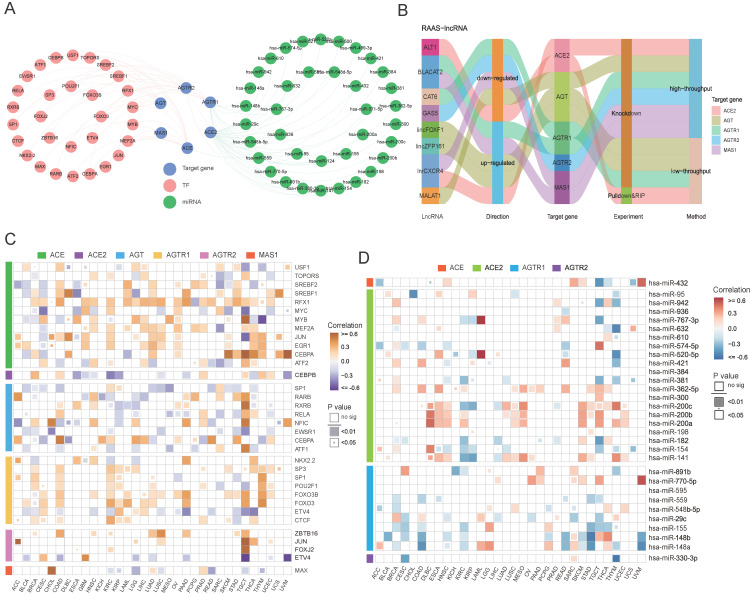
** Transcriptional and posttranscriptional regulatory mechanisms for RAS family. (A)** Interaction network of TF-RAS-miRNA from RegNetwork. Pink nodes stand for TF, green for miRNA, blue for target genes-RAS members. **(B)** Sankey plot for lncRNA-RAS interaction from LncRNA2Target database. **(C)** Heatmap for expression correlation between TF and RAS in TCGA 33 pan-cancers, evaluated by Pearson's coefficient. Yellow for positive correlation, blue for negative correlation. **(D)** Heatmap for expression correlation between miRNA and RAS in TCGA 33 pan-cancers, evaluated by Pearson's coefficient. Red for positive correlation, blue for negative correlation.

In addition to posttranslational regulation, transcriptional and posttranscriptional regulations of gene expression were explored. A transcription factor-RAS family network was constructed based on the RegNetwork database (**Figure 4A**), and particular relationships of RAS family with potential target genes, TFs (transcription factors) and miRNAs were displayed. Notably, RAS family members were correlated with the corresponding TFs and miRNAs (**Figure 4C**-**D**). ACE had a significant moderate positive correlation with CEBPA in testicular germ cell tumors (TGCT; Cor = 0.598), uveal melanoma (UVM; Cor = 0.638) and skin cutaneous melanoma (SKCM; Cor = 0.529). A positive association between CEBPA and AGT was observed in COAD (Cor = 0.502) and READ (Cor = 0.421). AGTR2 was positively related with JUN, a common transcription factor promoting tumor proliferation in both adrenocortical carcinoma (ACC; Cor = 0.614) and TGCT (Cor = 0.589). MAS1 was correlated with MAX in CHOL (Cor = 0.750), suggesting that MAX might play a principal role in the transcription of MAS1.

We integrated miRNA-target gene data from the RegNetwork, which includes both experimentally validated pairs from miRTarBase, TarBase, and miRecords and predicted interactions from miRanda, TargetScan, PicTar, MicroCosm, and micorT. Next, a miRNA-RAS family network was generated, which included 37 miRNAs targeting ACE, ACE2, AGTR1, and AGTR2 individually. The expression levels of miRNA-RAS factor pairs in 33 TCGA cancers were assessed. ACE2 and AGTR1 were most likely to be regulated by miRNAs because they contained the most miRNA binding sites. Notably, ACE was negatively correlated with hsa-miR-432 (Cor = -0.46) in TGCT, whereas ACE2 was negatively correlated with hsa-miR-632 (Cor = -0.50) in THYM. In addition, AGTR1 was maximally inhibited by hsa-miR-148b (Cor = -0.54) in STAD, and AGTR2 was maximally inhibited by hsa-miR-330-3p (Cor = -0.56) in THYM.

For posttranscriptional regulation, in addition to miRNAs, possible strategies involving lncRNAs targeting RAS family were also explored. Regulatory mechanisms of lncRNA-RAS family pairs were predicted by LncRNA2Target2.0, with the aim of providing candidates for functional studies (**Figure 4B**). MALAT1 was reported to be highly expressed in colon cancer and to contribute to tumor progression 42-44. The higher expression of AGT in COAD suggested that MALAT1 might promote tumor development through AGT. BLACAT2 is upregulated in bladder cancer 45, gastric cancer 46, and colon cancer 47 and negatively mediates the expression of AGTR1 and MAS1.

### Genomic alterations and fusion genes related to RAS family in pan-cancer

**Figure 5:**
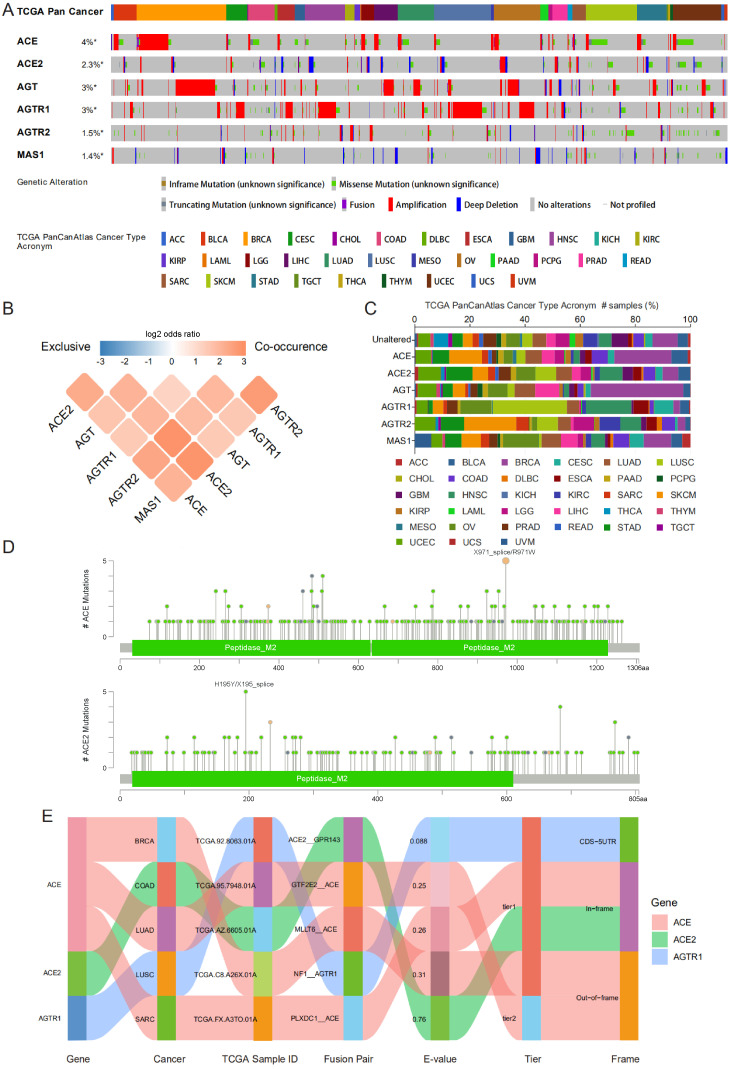
** Genomic alteration of RAS family in TCGA pan-cancer. (A)** Oncoprint for genetic alterations in RAS in TCGA pan-cancer from cbioportal. Each vertical bar points to each sample. **(B)** Heatmap for co-occurrence of RAS family in genomic alteration. **(C)** Alteration distribution of RAS family across TCGA pan-cancer. **(D)** Hotspot for amino acid mutation of ACE and ACE2 across TCGA 33 cancers. **(E)** Sankey plot for fusion genes of RAS menbers in diverse TCGA cancers.

Genomic alteration data containing mutation profile and copy number alteration (CNA) data related to RAS family were acquired from cBioPortal based on TCGA PanCancer Atlas program. In total, 10,967 samples from 32 types of cancer were analyzed. The oncoprint displayed an overall alteration frequency of RAS family of 15.2% in TCGA pan-cancer across TCGA cancers, with the highest frequency existing for ACE (4%), followed by AGT (3%), AGTR1 (3%), ACE2 (2.3%), AGTR2 (1.5%) and MAS1 (1.4%) (**Figure 5A**). ACE and AGT harbored the highest rates of genomic alterations in BRCA, ACE2 in UCEC, AGTR1 harbored the highest rates in LUSC, AGTR2 harbored the highest rates in SKCM, and MAS1 harbored the highest rates in OV (**Figure 5C**). The classical axis of the RAS family (involving ACE and AGTR1) was dominantly associated with amplification whereas the alternative axis of the RAS (involving ACE2 and MAS1) was associated with deletion in the CNA data. Moreover, a co-occurrence of genomic alterations was identified among RAS family (P<0.05). The higher the log2 odds ratio, the higher the coexistence tendency was (**Figure 5B**) based on CCLE data (**Supplementary Figure 4A**).

In all the TCGA pan-cancer samples, RAS family members differed in the frequency of missense mutations (**Figure 5D and Supplementary Figure 3**). Two samples with R971W and 3 samples with X971_splice mutations in ACE were observed. Five mutations in ACE2 were identified (2 X195_splice and 3 H195Y mutations), whereas 4 mutations in AGT were identified (all F430Lfs*25 mutations). The R167Q missense mutation in AGTR1 was found in 4 samples. In addition, in AGTR2, R155I and E291K missense mutations were identified in 4 samples, and R182* nonsense mutations were identified in 4 samples. Based on CCLE data, missense mutations were the most frequent alterations (**Supplementary Figure 4B**). A total of 10 samples harbored F430Lfs*25 FS del (n=8) and E431* FS ins (n=2) mutations in *AGT*.

The TCGA Fusion Gene Database was explored for further genomic alterations (**Figure 5E**). Three fusion genes were detected: *ACE-MLLT6*, *ACE-GTF2E2*, and *ACE-PLXDC1* in BRCA, LUAD, and SARC, respectively. NF1 is a classic tumor suppressor gene whose mutation can promote lung cancer 48, 49. Considering the low expression of AGTR1 in LUSC, the *NF1-AGTR1* fusion gene might serve as a new therapeutic target.

### Methylation and clinical importance of RAS family in pan-cancer

**Figure 6:**
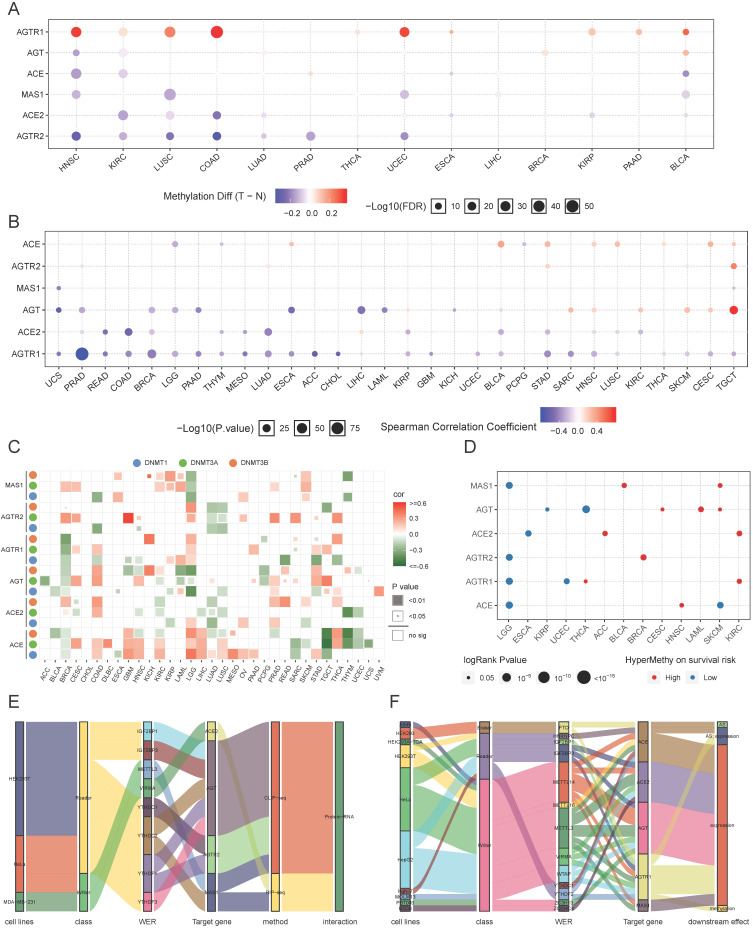
** Methylation of RAS family in TCGA pan-cancer. (A)** Distribution map of differential methylation levels between normal and tumor tissues in TCGA cancers. Blue points represent downregulation of methylation in tumors; red points represent upregulation of methylation in tumors. Data were analyzed by student T tests. The P value was adjusted by FDR, a FDR < 0.05 was considered as significant. **(B)** Correlation map for methylation and mRNA gene expression based on Spearman's coefficient. The P value was adjusted by FDR, only those genes with FDR < 0.05 were exhibited. **(C)** Bubble map of correlation of major DNA methyltransferases (DNMT1, DNMT3A, DNMT3B) and RAS family in TCGA 33 cancers, based on Pearson's coefficient. **(D)** Methylation survival of RAS estimated by Cox regression, only those genes with FDR < 0.05 were exhibited. Red dots indicate high survival risk of hypermethylation group, blue dots indicate low risk. **(E)** Sankey plot for binding map of m6A modification for RAS in cell lines. WER represents writers, erasers, readers respectively. **(F)** Sankey plot for perturbation map of m6A modification for RAS in cell lines. Downstream effects stand for different variations followed by perturbation of WER, including gene expression level, m6A level, translation efficiency and alternative splicing.

The genome is considered to be affected by one conventional epigenetic alteration, DNA methylation. The DNA methylation level of RAS family in TCGA data from 33 cancers was extracted using GSCALite. In contrast to other members, AGTR1 was distinctly hypermethylated in multiple cancers, such as COAD, head and neck squamous cell carcinoma (HNSC), UCEC, LUSC, and BLCA (**Figure 6A**), supporting previous studies in COAD 50 and LUSC 51. ACE2 was hypomethylated in COAD, KIRC, LUSC, LUAD, and KIRP. Differential methylation levels of RAS family members might be responsible for the differences in expression patterns between tumor tissue and normal tissues. To test this conjecture, the correlation of DNA methylation level with mRNA expression was assessed (**Figure 6B**). AGTR1 and ACE2 showed a negative correlation in most cancers.

Next, the regulation of the DNA methylation of RAS family was explored by specifically assessing the DNA methyltransferases: DNMT1, DNMT3A, and DNMT3B (**Figure 6C**). AGTR1 was negatively regulated by DNMT3A, DNMT3B, and DNMT1 in BRCA. AGT was negatively related to DNMT3A/DNMT3B in acute myeloid leukemia (LAML) and to DNMT1/DNMT3B in LGG. Since the expression of AGTR1 in BRCA and AGT in LAML/LGG was related to the DNA methylation level (**Figure 6B**), DNA methyltransferases might be critically involved in the DNA methylation process during tumor development and progression.

In the survival analysis (**Figure 6D**), hypermethylation of ACE, AGTR1, AGTR2, or MAS1 was associated with a lower survival risk in LGG, whereas hypermethylation of ACE2 or AGTR1 was associated with higher survival risk in KIRC. In the subsequent univariate survival analysis, ACE and AGTR1 predicted worse prognosis in LGG, whereas ACE2 and AGTR1 predicted better prognosis in KIRC.

In addition to DNA methylation, we assessed the potential regulation of m6A RNA methylation related to RAS family. M6A RNA methylation was found to be mainly involved in regulating the metabolic processes of RNA through three types of enzyme systems (WERs), thus affecting tumor development and progression. The m6A RNA methylation data of RAS family were extracted from m6A2Target, a comprehensive curated database including data on target genes of WERs focused on m6A modification derived from low-throughput experiments and high-throughput sequencing. Stratified of the factors by binding capability (**Figure 6E**) showed that AGT could interact with five readers (IGF2BP1, IGF2BP3, YTHDC2, YTHDF1, and YTHDF3) in HEK293T and HELA cell lines, as validated by CLIP-seq. AGTR2 could interact with two readers (YTHDC1 and YTHDF1). Moreover, MAS1 could interact with the METTL3 reader and the YTHDC2 writer. However, ACE2 might only interact with VIRMA in MDA-MB-231 cells. In the m6A WER assessment (**Figure 6F**), writers accounted for the majority of WERs targeting RAS family. The influence of m6A methylation on RAS family was mostly a result of effects on gene expression, which is closely related to alternative splicing (AS) and methylation.

### Association of clinical phenotypes and RAS family

**Figure 7:**
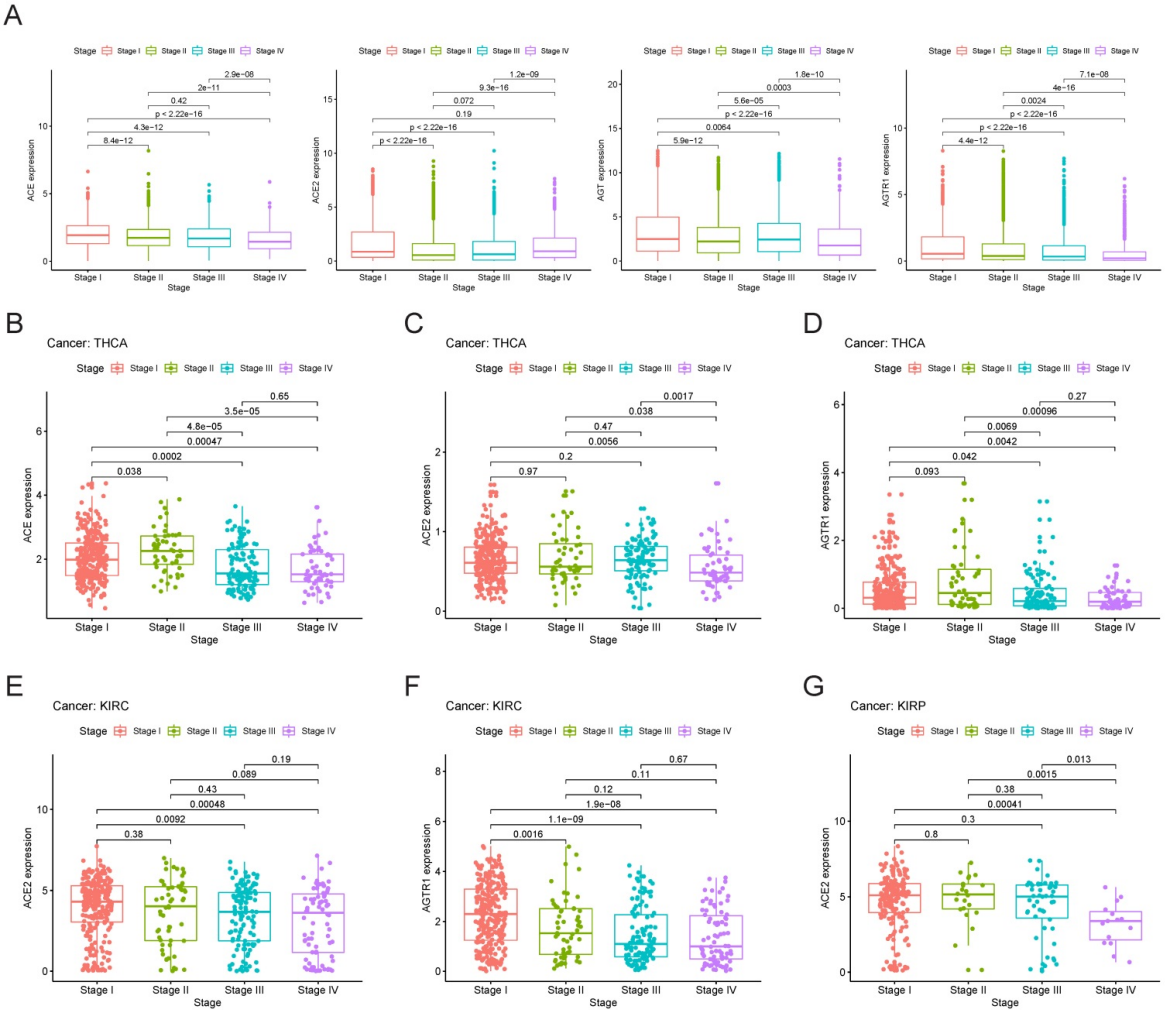
** Clinical stages of TCGA cancers and gene expression patterns of RAS family. (A)** Expression of ACE, ACE2, AGT and AGTR1 in distinct clinical stages of pan-cancer. Expression of **(B)** ACE, **(C)** ACE2 and **(D)** AGTR1 in distinct clinical stages of THCA. Expression of **(E)** ACE2 and **(F)** AGTR1 in distinct clinical stages of KIRC. **(G)** Expression of ACE2 in distinct clinical stages of KIRP.

Initially, we explored the expression of RAS family members in different stages of TCGA pan-cancer (**Figure 7A**). The expression levels of AGTR1 and ACE decreased with increasing stage. ACE2 expression was decreased in stage II cancer but gradually increased in stage III and IV cancers. Despite augmented expression in stage I and III cancers, AGT was decreased in stage II and IV cancers. However, the inclusion of all types of tumor samples may have prevented the discovery of an authentic expression trend. Therefore, we performed independent analyses of each tumor type and compared their expression levels in subgroups. Specific associations between gene expression and clinical stage were explored in THCA, KIRC, and KIRP (**Figure 7B-G**). In THCA, the expression of ACE and AGTR1 was decreased in stage III/IV compared to stage I/II cancer. Compared to stage I cancer, stage IV cancer showed a decrease in ACE2 in THCA. Decreases in the expression of ACE2 and AGTR1 in KIRC were detected. In KIRP, ACE2 was reduced distinctly in stage IV cancer.

### Survival analysis of RAS family in pan-cancer

**Figure 8:**
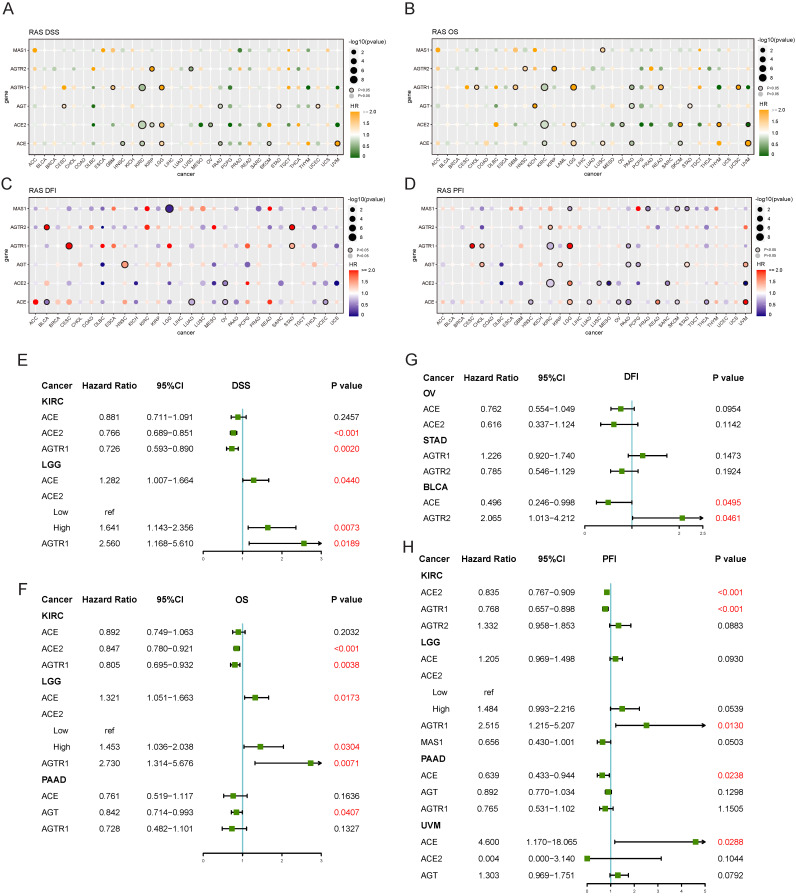
** Prognostic value of RAS family in TCGA pan-cancers.** Univariate analysis of **(A)** DSS, **(B)** OS, **(C)** DFI, **(D)** PFI of individual members of RAS in 33 cancers. Genes labeled with borders were significant (p<0.05). Multivariate analysis of **(E)** DSS, **(F)** OS, **(G)** DFI and **(H)** PFI of individual members of RAS in specific cancer, in which containing three or more genes of statistical significance.

Both univariate and multivariate Cox hazard ratio regression models were established. Four endpoints were analyzed in TCGA to determine the prognostic value of RAS family. RAS family was significant prognostic factor for disease-specific survival (DSS) in KIRC and LGG: increased expression of ACE, ACE2, and AGTR1 was a protective factor in KIRC but an adverse risk factor in LGG (**Figure 8A**). In subsequent multivariate analyses, ACE2 and AGTR1 were independent prognostic factors in both KIRC and LGG (**Figure 8E**). Similarly, ACE, ACE2, and AGTR1 were significant prognostic factors for overall survival (OS) in KIRC and LGG, whereas ACE, AGT, and AGTR1 were significant prognostic factors for OS in pancreatic adenocarcinoma (PAAD) (**Figure 8B**). ACE2 and AGTR1 were independent prognostic factors for OS in KIRC and LGG, whereas AGT was an independent factor in PAAD (**Figure 8F**). Although in DFI, RAS family had an individual impact in BLCA, OV, and STAD in the univariate analyses (**Figure 8C**), ACE and AGTR2 had only weak prognostic significance in BLCA in the multivariate analysis (**Figure 8G**).

Survival data on RAS family across GEO profiles from the PrognoScan database were included (**Supplementary Figures 5 & 6**). High expression of ACE suggested a poorer prognosis in hematologic tumors (GSE4475, E-Tabm-346) but a better prognosis in breast cancer and colorectal cancer. However, in ovarian cancer, the results were inconsistent for OS, and the confidence interval indicated that the results of the analysis of GSE26712 might be reliable. For RFS and DMFS in breast cancer and for DFS and DSS in colorectal cancer, AGT was a protective factor. And AGT was a risk factor for OS in LUAD, ovarian cancer, and melanoma. High expression of AGTR1 was correlated with better survival in breast cancer, LUAD, and liposarcoma. AGTR2 was a risk factor in follicular lymphoma and colorectal cancer, whereas MAS1 was a protective factor in most tumors.

### Functional prediction of RAS family in pan-cancer

**Figure 9:**
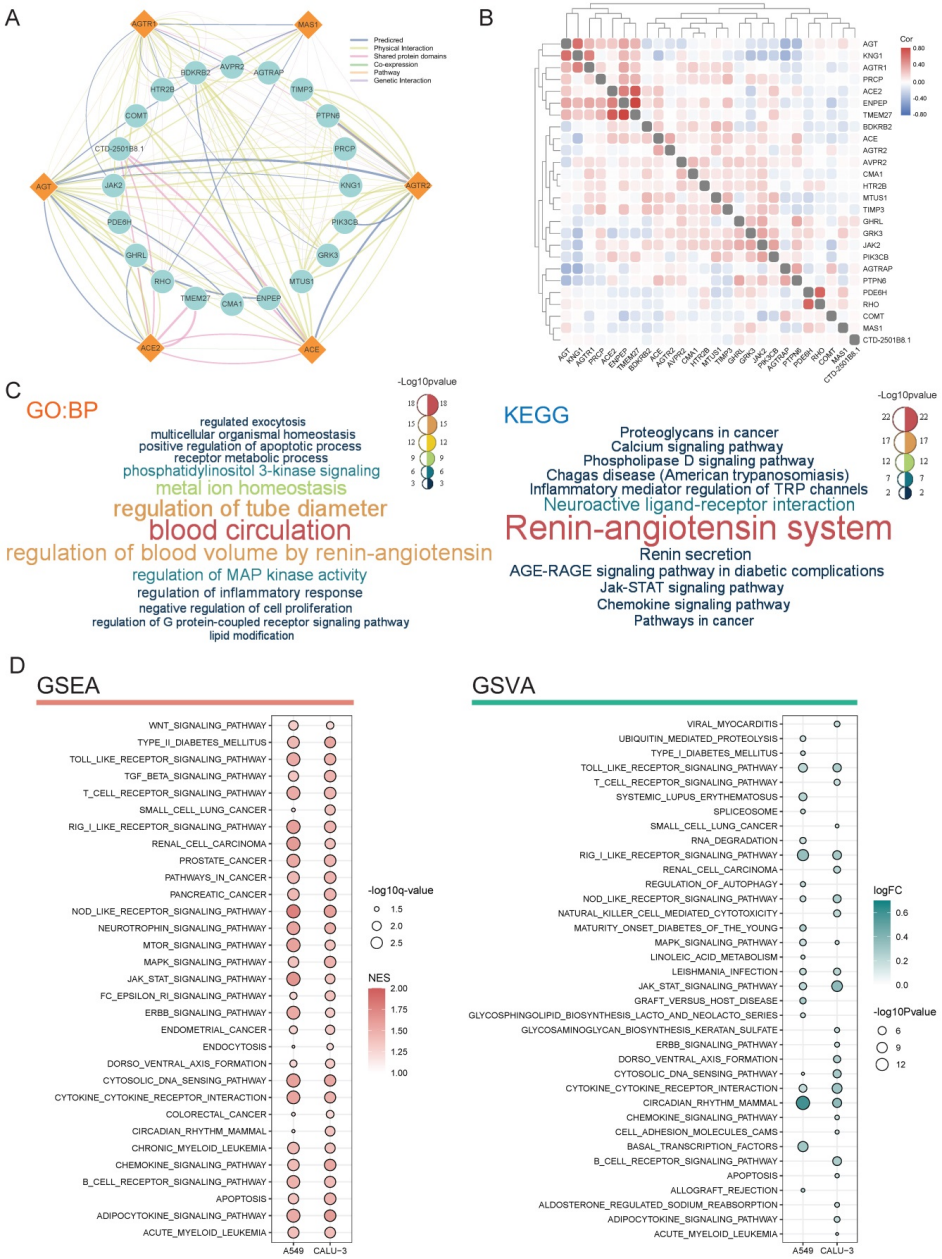
** Predicted functions of RAS family. (A)** Protein-protein-interaction network of RAS family assessed by GeneMANIA. **(B)** Heatmap for correlation between RAS family and interactive proteins across all tumors. **(C)** Word cloud of enrichment analysis of Biological process of Gene Ontology and KEGG pathway through RAS and interacting proteins. **(D)** Bubble plot for KEGG pathway enriched by GSEA and GSVA in calu-3 and A549 infected by SARS-CoV-2.

Using GeneMANIA, a protein-protein interaction network of RAS family was constructed for more in-depth functional analyses. The GeneMANIA prediction tool generates a biological network for gene prioritization and can be used to predict gene function (**Figure 9A**). Physical pairings accounted for the majority of interactions. The correlations of RAS family with interacting proteins were determined by Pearson's coefficient analysis of 33 TCGA cancers (**Figure 9B**). The associations between RAS family members were low, except for a weak positive correlation between ACE and ACE2. Both ACE and AGTR1 were positively related to KNG1. Thus, the classic axis of the RAS family might play a synergistic role with KNG1. A positive correlation between ACE2 and TEME27 was observed: low expression of both was associated with a high risk of death in renal clear cell carcinoma 52. Since ACE2 functions as a protective factor in prognosis in renal clear cell carcinoma, we speculated that ACE2 and TEME27 collaboratively inhibit the progression of renal clear cell carcinoma tumors.

GO enrichment analyses of biological process terms and KEGG pathways were performed to detect the potential functions of proteins interacting with RAS family (**Figure 9C**). Based on the GO and KEGG enrichment results, in addition to regulating blood circulation, RAS family were associated with multiple classic signaling pathways in tumor progression, such as the PI3K, MAPK, calcium ion, and JAK-STAT pathways. In addition, the biological process terms inflammatory response containing cytokines and chemokines, regulation of TRP channels, and JAK-STAT were enriched. In addition, RAS family was identified to be involved in metabolic processes.

By infecting the human respiratory tract through the ACE2 receptor on the cellular membrane, SARS-CoV-2 causes a severe inflammatory response and damage to various organs (such as the lung, heart, kidney, colon). Deletion of ACE2 may cause such a cytokine storm after binding to SARS-CoV-2, which leads to accumulation of Ang II and activation of the classical axis through binding of AGTR1. Here, using GSEA and GSVA, possible effects of SARS-CoV-2 infection on tumor cells were explored based on RNA-seq results (**Figure 9D**). The lung cancer cell lines Calu-3 and A549 overexpressing ACE2 39 were infected with SARS-CoV-2 (GSE147507). GSEA identified activation of pathways related to various cancers (small-cell lung cancer, renal cell cancer, prostate cancer, pancreatic cancer, colon cancer, and leukemia) in response to SARS-CoV-2 infection. Moreover, several common cancer-related pathways, including the WNT, TGF-β, mTOR, MAPK, JAK-STAT, and ERBB pathways, were also activated.

More importantly, many pathways involved in immune inflammation were identified, in particular, the T cell receptor, B cell receptor, cytokine receptor, and JAK-STAT pathways. Based on the GSVA, SARS-CoV-2 infection was associated with immune-related, cancer, and downstream pathways. Notably, functional pathways involved in the RAS family overlapped with those involved in SARS-CoV-2 infection. We speculated that SARS-CoV-2 infection might affect the biological processes of tumor development and progression partially by interacting with the RAS family in the respiratory and circulatory systems.

### Association of immune infiltration and RAS family in pan-cancer

**Figure 10:**
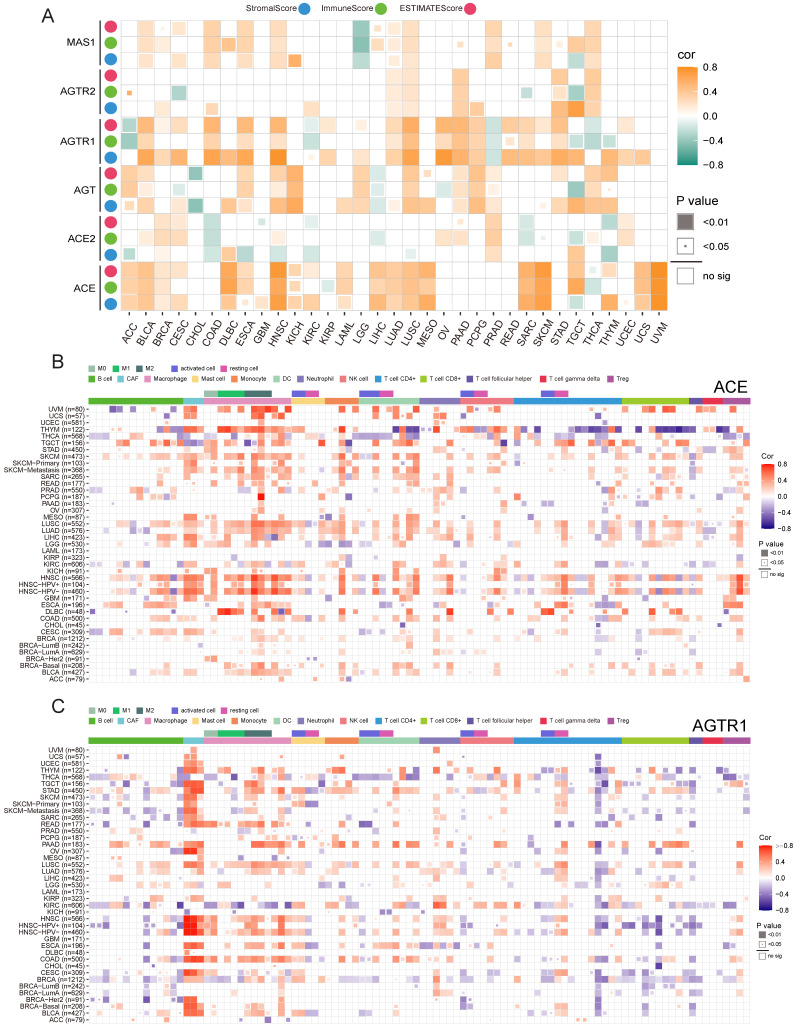
** Immune infiltration and expression of RAS family. (A)** Heatmap for correlation between ESTIMATE score and mRNA expression of RAS family across TCGA 33 cancers. Blue, green or pink point indicates Stromal, Immune and ESTIMATE score (combination of Stromal and Immune score), respectively. Heatmap for correlation between immune cell infiltration and mRNA expression of **(B)** ACE and **(C)** AGTR1 integrated with multiple databases including CIBERSORT, TIMER, XCELL, EPIC, MCQUANTER, QUANTISIQ. All results were adjusted by tumor purity.

Previously, we discussed the functions of RAS family and possible downstream mechanisms underlying SARS-CoV-2 infection of tumor cells. The potential relevance of the RAS family to immune-inflammatory processes led us to explore its role in the tumor microenvironment (TME). We identified the association between RAS family and the ESTIMATEScore to assess immune infiltration level by quantifying stromal cells and immune cells in the tumor microenvironment (**Figure 10A**). In most tumors, ACE and AGTR1 (in the classical axis) possessed a closer correlation with non-tumor components than those members in the alternative axis and AGTR2. Moreover, the positive association between ACE and the ESTIMATEScore was the strongest in HNSC, SKCM, and UVM. AGTR1 expression was more related to stromal cells than to immune cells. The relationship of RAS family with immune cells was explored by integrating data from several databases (CIBERSORT, TIMER, xCell, EPIC, MCP-counter, and QUANTISIQ) currently available for assessing immune cell components (**Figure 10B,C & Supplementary Figure 7**). Corresponding to our findings for the ESTIMATEScore, ACE expression was positively associated with immune cells in HNSC, SKCM, LIHC, LUSC, LUAD, or UVM. Notably, ACE expression was strongly connected with macrophages and cancer-associated fibroblasts (CAFs) in most tumors and was more related to M2-type macrophages. Regulatory T cells (Tregs) were highly related to ACE expression in HNSC. AGTR1 expression was similar to that of ACE and was positively related to CAFs and macrophages. Thus, the classic axis of the RAS family has an impact on macrophages and CAFs in the TME.

### Association between immunotherapy and RAS family in pan-cancer

**Figure 11:**
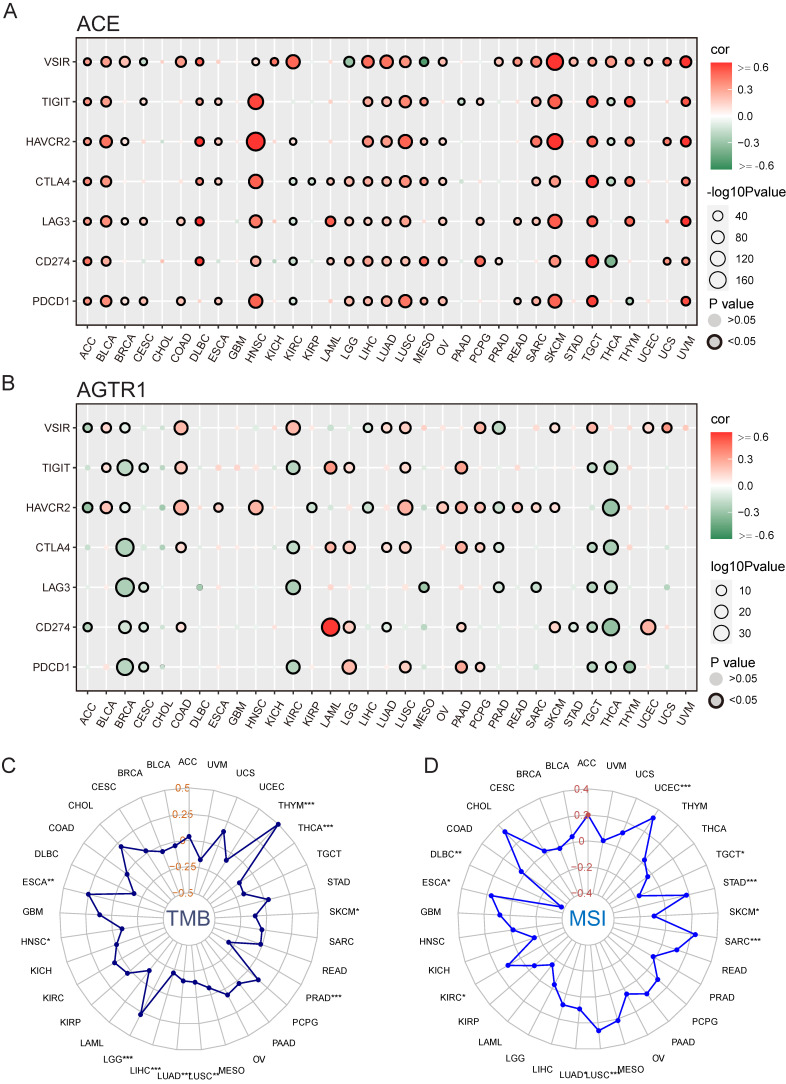
** Potential role of ACE in immunotherapy in pan-cancers. (A)** Bubble plot for correlation between expression of major immune checkpoint genes and ACE in TCGA 33 cancers. **(B)** Bubble plot for correlation between expression of major immune checkpoint genes and AGTR1 in TCGA 33 cancers. Radar maps for correlation between **(C)** TMB, **(D)** MSI and ACE expression under TCGA pan-cancers. *P < 0.05, **P < 0.01, ***P < 0.001.

We hypothesized that ACE and AGTR1 might play roles in interacting with the TME and thus regulating the response to immunotherapy. We determined the relationships of ACE and AGTR1 with signature immune checkpoint genes (PDCD1, CD274, LAG3, CTLA4, HAVCR2, TIGIT, and VSIR/VISTA). ACE was related to immune checkpoint genes across pan-cancer, particularly HNSC, LUSC, SKCM, TGCT and UVM (**Figure 11A**). The findings were in agreement with the immune infiltration analysis results and expression of ACE in tumors. In contrast, AGTR1 was only strongly associated with CD274 (PD1) in LAML (**Figure 11B**). Therefore, we hypothesized that the combination of ACE inhibitors (ACEIs) with immunotherapy might result in better outcomes.

TMB and MSI are innovative biologic candidates for assessing PD1/PD-L1 immunotherapy response and have been applied in clinical practice and trials. Here, ACE was positively associated with TMB in THYM (**Figure 11C**). In addition, ACE was positively related to MSI in UCEC and CHOL (**Figure 11D**). These findings indicate the biological relevance of ACEIs in PD1/PD-L1 treatment.

## Discussion

To deeply elucidate the roles of RAS family, a comprehensive analysis of multiomics and multifaceted information on expression distribution and regulation, genomic and epigenetic alterations, clinical phenotypes and immune infiltration was performed. The findings could provide new insights into the involvement of SARS-CoV-2 in carcinogenesis.

Previous studies have demonstrated that SARS-CoV can downregulate ACE2 but upregulate Ang II expression after entering cells through ACE2, activate the downstream AGTR1 receptor, and subsequently cause the release of various inflammatory factors, leading to severe acute respiratory distress syndrome (ARDS) 6, 53. SARS-CoV-2 and SARS-CoV, β coronaviruses, enter host cells through the ACE2 receptor on the host cellular surface. In addition, an increase in plasma Ang II is linearly associated with viral load and lung injury in COVID-19 patients 5. Decreased ACE2 expression and harmful accumulation of Ang II after SARS-CoV-2 infection might initiate imbalance of the RAS family and damage to target organs. Importantly, imbalance of the RAS family might play a vital role in tumor development and progression. It remains to be determined whether SARS-CoV-2 can aggravate this imbalance after infecting the human body and produce biological effects on tumors. Earlier studies on the RAS family were mostly limited to specific tumors and had a limited number of patients. They focused on changes in a single gene, ignoring the potential roles of other genes and the whole system. In both normal physiological and pathological states, each member of the RAS is interrelated, coordinated, and mutually restricted. Therefore, we aimed to reveal the expression regulation mechanisms of the RAS family in cancer through multidimensional and multifaceted analyses of 6 primary RAS members and to provide a basis for the study of SARS-CoV-2 in cancer patients. Other novel members of the RAS, such as the (pro) renin receptor (PRR), were studied recently. PRR could activate the RAS by binding to renin but also activate downstream signaling pathways independent of the RAS system 54. Studies have confirmed the potential of PRR as a biomarker in renal clear cell carcinoma 55. Therefore, it was necessary to further study its role in the RAS family.

An overview of RAS family expression based on data from the GTEx and TCGA databases was performed for normal and tumor tissues. The expression levels of AGTR2 and MAS1 were deficient in all tissues, while ACE, ACE2, AGT, and AGTR1 were universally expressed in most tissues. ACE2 and ACE were abundantly expressed in the small intestine and testicular tissue, consistent with recent reports 56-58, and this high expression might lead to more severe Ang II accumulation in the intestinal and testicular tissues than in other tissues. The high levels of ACE2 and ACE in testicular tissue may indicate that male testicular tissue might be more vulnerable to invasion by SARS-CoV-2 than are other organs. AGTR1 is one of the primary receptors binding Ang II, which acts as a downstream biological factor of the RAS. AGTR1 was found to be expressed in adipose tissue and the adrenal gland and mammary gland, suggesting that the classical axis has an impact on these tissues. The expression distribution of AGTR1 among tumors was similar to that in normal tissues. AGTR1 was expressed in ACC, LIHC, and corresponding cancer cell lines. AGTR2 and MAS1 were weakly expressed in normal and tumor tissues as well as cancer cell lines. ACE was highly expressed in CHOL compared with normal tissue, supported a previous study reporting that ACE was highly expressed in cancer patients in serum level 59.

ACE and ACE2 were highly expressed in tumors at the mRNA level, with differential expression at the protein level, as previously reported 60. ACE2 was highly expressed at the mRNA and protein levels in renal tumors, COAD and READ. Such results were consistent with those of recent studies focusing on ACE2 57, 61. Moreover, ACE2 expression was reported to be slightly higher in tumor than in normal tissues 20, but the difference was not statistically significant due to the limited sample size. ACE2 was highly expressed in colon cancer, suggesting that patients with colon cancer might be more susceptible to SARS-CoV-2 infection than healthy people. AGTR1 was weakly expressed in almost all tumors compared to normal tissues, inconsistent with previous reports. AGTR1 was reported to be highly expressed in tumors in the GENT database 62, and this high expression was associated with poor prognosis. AGTR1 was also highly expressed in ER^+^ breast cancer 63, pancreatic cancer 64, and gastric cancer 65. To validate the expression pattern of AGTR1, we investigated the ONCOMINE database (https://www.oncomine.org/resource/login.html), which includes data from multiple tumor types derived from expression profile chip analysis of many samples. In accordance with our results, AGTR1 was expressed at low levels in tumor tissues in 73 pairs from more than 14 tumor types but overexpressed in normal tissues in a set of prostate cancer pairs. Moreover, no significant differences were observed for cervical, esophageal, or gastric cancer or myeloma. This result implied that the differential expression identified based on the GTEx and TCGA data was reliable. In addition, AGTR1 inhibitors, such as candesartan 66, could effectively reverse the malignant behavior of tumors and prolong the survival time of patients. Although AGTR1 expression was lower in most tumor tissues than in normal tissues, it still played a role in tumor development.

Of particular concern was the inconsistent association between the mRNA and protein levels of ACE in KIRC and OV; ACE2 in KIRC and LUAD; and AGT in BRCA, COAD, and LUAD. This phenomenon suggests that more attention should be given to changes in RAS family induced by PTMs. In our assessment of an E3 ubiquitin ligase database, RAS family was found to bind various E3 ubiquitin ligases. Three E3 ligases (NEDD4, CBL, and SYVN1) positively associated with ACE may participate in ubiquitination after ACE translation in KIRC and OV, causing protein degradation. RAS family transcriptional and posttranscriptional modifications were also assessed. CEBPA was positively correlated with ACE and AGT expression in several tumors. Compared to their normal counterparts, colon adenocarcinoma (COAD) and READ might feature positive transcriptional regulation of CEBPA, which is highly expressed in colon cancers to promote malignant biological properties 67. The negative relationship of AGTR1 with hsa-miR-148b and hsa-miR-148a in COAD, STAD, and UVM indicated a dominant impact of hsa-miR-148 on the posttranscriptional modification of AGTR1.

Comprehensive analysis of the expression of RAS family in tumors suggested that patients with tumors and high expression of ACE2 (e.g., those with COAD or READ) might be susceptible to SARS-CoV-2 infection. However, we should also note that ACE was highly expressed in CHOL, KIRC, and PCPG. When patients with such tumors had SARS-CoV-2 infection, the secondary RAS family imbalance could produce further activation of the ACE-Ang II-AGTR1 axis and possibly exacerbate the progression of the original disease.

In the genomic alteration analysis, RAS family members tended to be closely linked to each other and to function cooperatively. In addition, the classic and alternative axes of the RAS family exhibited inconsistent alteration trends in copy number variation. The classic axis members ACE and AGTR1 tended to be amplified. In contrast, the alternative axis members ACE2 and MAS1 harbored more deletions/insertions, which explains their opposite biological effects from a genomics point of view. Abnormal upregulation of the classic axis factors caused by amplification and downregulation of the alternative axis factors caused by deletions/insertions might be involved in tumor development. Epigenetic modification, represented by DNA methylation, of RAS family was explored. AGTR1 was hypermethylated in COAD, HNSC, UCEC, LUSC, and BLCA, as previously reported 50, 51. DNA methylation is involved in regulating the mRNA expression of RAS family. Subsequently, the correlation of DNA methylation was with survival was assessed. Interestingly, hypermethylation of ACE, AGTR1, AGTR2, or MAS1 in LGG was associated with longer survival time, while hypermethylation of ACE2 or AGTR1 in KIRC was related to shorter survival time. These findings supported the results of our univariate survival analyses of RAS family. Thus, DNA methylation of RAS family regulates the biological processes of cancer.

Significant expression differences between clinical stages were observed only in THCA, KICH, and KIRP. In THCA, ACE, ACE2, and AGTR1 decreased with increasing clinical stage. The expression levels of ACE2 and AGTR1 in KIRC were lower in stage II, III, and IV disease (without significant differences among them) than in stage I disease. As previously reported 60, 68, there was no significant association between the expression levels of ACE2 and clinical stage in renal clear cell carcinoma; therefore, additional investigation is needed to confirm the effects of ACE2 expression on KIRC stage. Based on Cox hazard ratio regression models, RAS family was found to have an influence on the survival of KIRC, LGG, and PAAD. Previously, ACE2 was reported to be unrelated to the prognosis of renal clear cell carcinoma 68. However, among over 500 TCGA samples, ACE2 and AGTR1 were found to be related to OS or DSS, and therefore, they might be used as independent prognostic factors for KIRC; in contrast, ACE2 was related to OS in the E-DKFZ-1 cohort (including data from transcription profiling of 74 kidney tumor samples of different histological types, differentiation grades, and stages and data on chromosomal aberrations and follow-up). Similar results were observed in other pancancer studies of ACE2 based on TCGA data, in which ACE2 was found to be a potential prognostic factor for kidney cancer 56, 61, 69. Several studies identified the ACE I/D polymorphism that is strongly related to the prognosis of glioma 70, 71. ACEI and AGTR1 inhibitors could prolong the OS and PFS of glioma patients 20. In our study, AGTR1 expression correlated with OS and DSS in GBM. We utilized multivariate Cox regression models for the first time to establish ACE, ACE2, and AGTR1 as independent risk factors for LGG. AGTR1 expression was significantly associated with LGG progression-free interval (PFI). Thus, more attention should be given to the roles of RAS family in LGG.

Previous studies have provided evidence for the pathophysiological roles of RAS family in a variety of tumors through many downstream pathways. The Ang II-AGTR1 pathway can activate downstream ERK/MAPK 72, 73 and PI3K 74, 75 signaling and interact with different receptor tyrosine kinases to promote tumor development and progression. Activated AGTR1 also recruits nonreceptor tyrosine kinases such as SRC to activate JAK-STAT pathways 76. The Ang II-AGTR1 pathway participates in cytoskeletal contraction regulation and extracellular matrix production to induce epithelial-mesenchymal transition (EMT) in cancer cells 77, which is intimately related to tumor invasion and metastasis. The Ang II-AGTR1 signaling can stimulate the expression of cytokines and growth factors, triggering a multiple downstream signaling cascades that ultimately lead to the recruitment of immune cells. However, the Ang II-AGTR2 pathway induces tumor suppression by inhibiting cell proliferation and concurrently stimulating cell apoptosis in a variety of cells 78, 79. The ACE2-Ang (1-7)-MAS1 axis exists as an antagonist of the classical axis, playing a protective role in tumorigenesis. We identified RAS system downstream functions and pathways involved in processes in pan-cancer by GO and KEGG enrichment analyses of proteins interacting with RAS family. In addition, we performed GSEA and GSVA of SARS-CoV-2 infected lung cancer cell lines. After virus infection, tumor cells upregulated immune-inflammatory-related pathways and cancer-related pathways, and there was much overlap between the pathways upregulated by SARS-CoV-2 and those upregulated by RAS family. SARS-CoV-2 infection of host cells could lead to decreased ACE2 expression, imbalance of the RAS system, and toxic accumulation of Ang II, thus triggering inflammatory storms. Consequently, we speculated here that SARS-CoV-2 infection of tumor cells might enable downstream functions through the RAS system, potentially enhancing malignant behavior.

As an essential factor initiating tumor development, inflammation, and concomitant immune responses, the RAS family plays a substantial role in the TME. Tregs, myeloid-derived suppressor cells (MDSCs), M2-type macrophages, and CAFs, as well as the bioactive components they produce, comprise an immunosuppressive TME, inhibit the anti-tumor immune response, and promote tumor development and progression. We found that ACE was positively correlated with M2-type macrophages in most tumors. In contrast, AGTR1 was linked to CAFs. Ang II promoted the expansion of tumor-associated macrophages (TAMs) and the growth of non-small-cell lung cancer cells 80. ACEI treatment inhibited the expansion of TAM progenitor cells and reversed the growth of tumors. Ang II reduced CD8^+^ cell infiltration by increasing the infiltration of Tregs, fibroblasts, and macrophages, leading to an immunosuppressive TME in a 4T1 cell-generated breast cancer model 81.

Next, we explored the association between the RAS family and immunotherapy in carcinomas. ACE positively correlated with immune checkpoint molecules in almost all cancer types. The Ang II-AGTR1 axis induced an inhibitory immune TME by upregulating PD-L1 in non-small-cell lung cancer 82. In a mouse model of colon cancer, angiotensin II receptor blockers (ARBs) significantly reduced inhibitory T cells and a variety of immunosuppressive factors, including IL-6, IL-10, and VEGF. ARBs also decreased CAFs and immunosuppressive factors, such as CCL12 and NOS2 83. All these findings suggest that ACEIs/ARBs might achieve better efficacy in combination with immune checkpoint inhibitors (ICIs). Moreover, although the association between ACE and PD-L1/PD-1 in THYM is not strong, for the first time, we observed a moderate correlation between ACE and TMB in THYM, indicating that the application of ACEIs in THYM might bring benefits to patients with a high TMB. Zhang et al. found that ACE2 was positively correlated with the immune-promoting/immunosuppressive cell ratio in a variety of cancers and was associated with the anti-PD-1/PD-L1/CTLA-4 immunotherapy response. This finding suggests that the ACE2-Ang(1-7)-MAS1 axis plays an immune-promoting role in the TME 84.

The three most vital immunological manifestations of COVID-19 are lymphocytosis, overexpression of immune checkpoints, such as PD-1, PD-L1, and Tim-3, and excessive release of cytokines by monocytes and neutrophils 85. We also noted that there was no increase in SARS-CoV-2 infection-related mortality in cancer patients who had recently been treated with ICI therapy 86. During the COVID-19 pandemic, one older inpatient with multiple comorbidities was treated with nivolumab (a PD-1/PD-L1 inhibitor) for metastatic malignant melanoma and was in good condition, and no pneumonia was detected 87. As we mentioned above, the combination of ACEIs/ARBs and ICIs may benefit cancer patients. Therefore, considering the imbalance of the RAS family (overactivation of the ACE-Ang II-AGTR1 axis) in COVID-19, a combination of ACEIs/ARBs and ICIs could be considered for use in cancer patients infected with SARS-CoV-2.

In addition to the respiratory system, the cardiovascular system is also damaged by SARS-CoV-2. It has been reported that patients with COVID-19 combined with cardiovascular diseases (CVDs), such as hypertension, diabetes, and coronary heart disease, have more severe clinical manifestations and higher mortality 88. There are many common pathological mechanisms between cancers and CVDs, and the RAS system is also similar between these diseases. The systemic RAS was overactivated during heart failure, with increased release and activity of Ang II in the blood and elevated plasma aldosterone levels 89. Therefore, in tumor patients infected with SARS-CoV-2, the downregulation of ACE2 levels might cause an imbalance in the RAS system and activate the ACE-Ang II-AGTR1 axis, which would likely aggravate the clinical symptoms of tumor patients with heart disease and even induce heart failure. Although the use of RAS inhibitors such as ACEIs/ARBs has been shown to increase the expression of ACE2 in some animal studies 90, 91, the use of RAS inhibitors has been recommended in CVD patients with COVID-19 since there has been no clinical evidence that the use of RAS inhibitors increases the susceptibility to SARS-CoV-2 infection 92, 93. However, more clinical evidence is needed to confirm whether ACEI/ARB use should be continued in cancer patients with underlying heart disease who was infected with SARS-CoV-2.

Since the outbreak of COVID-19, many studies have focused on ACE2, the main receptor of SARS-CoV-2 58, 94. However, such studies have ignored the overall role of the RAS family in cancer and COVID-19. Our study focused on the RAS family as a whole and was not limited to ACE2. By incorporating GTEx data, our work overcame the absence of normal samples as controls for TCGA tumors. We supplemented survival analyses of RAS family in several common tumors with GEO data. We compared the expression differences between various types of tumors after removing non-tumor components with the use of tumor cell lines. All analyses were based on 33 types of tumors. Nevertheless, a major limitation was the lack of experimental validation *in vitro* or *in vivo*. Some rare tumors in TCGA (such as ACC, CHOL, MESO, pheochromocytoma and paraganglioma [PCPG], uterine carcinosarcoma [UCS], and UVM) had very small sample sizes and required substantial investigation in the near future. In addition, although the protein expression patterns of RAS family were discussed, there was a lack of survival information, as the protein is the ultimate unit that performs the biological functions. In addition, large-scale clinical trials are needed in the future to verify the effects of RAS inhibitors on cancer patients with SARS-CoV-2 infection.

## Conclusion

This work has demonstrated a complex and comprehensive landscape of the RAS family in oncology. The RAS family exists in most human tissues as well as various tumor tissues. Differential expression of RAS family existed across TCGA pan-cancer dataset. Potential prognostic effects of ACE2 and AGTR1 expression were associated with DNA methylation status in LGG and KIRC. ACE and AGTR1 expression were positively related to immunosuppressive components in the TME and were highly associated with immune checkpoints and immunotherapy indicators such as MSI/TMB. SARS-CoV-2 might affect tumor development and progression by affecting variant expression distribution and biologic/pathologic functions of the RAS. ACEIs/ARBs (ACE/ANG II/AGTR1 axis inhibitors) might benefit cancer patients with SARS-CoV-2 infection.

## Supplementary Material

Supplementary figures and table.

## Abbreviations

ACC: Adrenocortical carcinoma
ACE: Angiotensin converting enzyme
ACE2: Angiotensin converting enzyme 2
AGT: Angiotensinogen
AGTR1: Angiotensin II receptor 1
AGTR2: Angiotensin II receptor 2
ARDS: Acute respiratory distress syndrome
BLCA: Bladder urothelial carcinoma
BRCA: Breast invasive carcinoma
CAFs: Cancer-associated fibroblasts
CCLE: Cancer Cell Line Encyclopedia
COAD: Colon adenocarcinoma
CHOL: Cholangiocarcinoma
CPTAC: Clinical proteomic tumor analysis consortium
DFI: Disease free interval
DMFS: Distance metastases-free survival
DSS: Disease specific survival
GBM: Glioblastoma multiforme
GO: Gene ontology
GSEA: Gene set enrichment analysis
GSVA: Gene set variation analysis
GTEx: Genotype-Tissue Expression
HNSC: Head and Neck squamous cell carcinoma
KEGG: Kyoto Encyclopedia of Genes and Genomes
KICH: Kidney chromophobe
KIRC: Kidney renal clear cell carcinoma
KIRP: Kidney renal papillary cell carcinoma
LAML: Acute myeloid leukemia
LGG: Brain low grade glioma
LncRNA: Long-noncoding RNA
LUAD: Lung adenocarcinoma
LUSC: Lung squamous cell carcinoma
LIHC: Liver hepatocellular carcinoma
MAS1: Mas1 receptor
MDSC: Marrow-derived suppressor cells
MESO: Mesothelioma
MSI: Microsatellite instability
NSCLC: Non-small cell lung cancer
OS: Overall survival
OV: Ovarian serous cystadenocarcinoma
PAAD: Pancreatic adenocarcinoma
PCPG: Pheochromocytoma and Paraganglioma
PFI: Progression free interval
PPI: Protein-protein interaction
RAS: Renin-angiotensin-system
READ: Rectum adenocarcinoma
RFS: Recurrence free survival
SARS-CoV: Severe acute respiratory syndrome coronavirus
SARS-CoV-2: Severe acute respiratory syndrome coronavirus-2
SKCM: Skin cutaneous melanoma
STAD: Stomach adenocarcinoma
TCGA: The Cancer Genome Atlas
TF: Transcription factor
TGCT: Testicular germ cell tumors
THCA: Thyroid carcinoma
THYM: Thymoma
TICs: Tumor infiltration cells
TMB: Tumor mutation burden
TME: Tumor microenvironment
UCEC: Uterine corpus endometrial carcinoma
UCS: Uterine carcinosarcoma
UVM: Uveal melanoma
WER: Writers/Erasers/Readers
