# Supplementary material for: Comprehensive landscape of the renin-angiotensin system in Pan-cancer: a potential downstream mediated mechanism of SARS-CoV-2
Source: Int J Biol Sci. 2021 Sep 3;17(14):3795–817. doi: 10.7150/ijbs.53312 (PMC8495399; doi:10.7150/ijbs.53312)
Supplement: Supplementary file 1 — Supplementary figures and table. [file ijbsv17p3795s1.pdf]

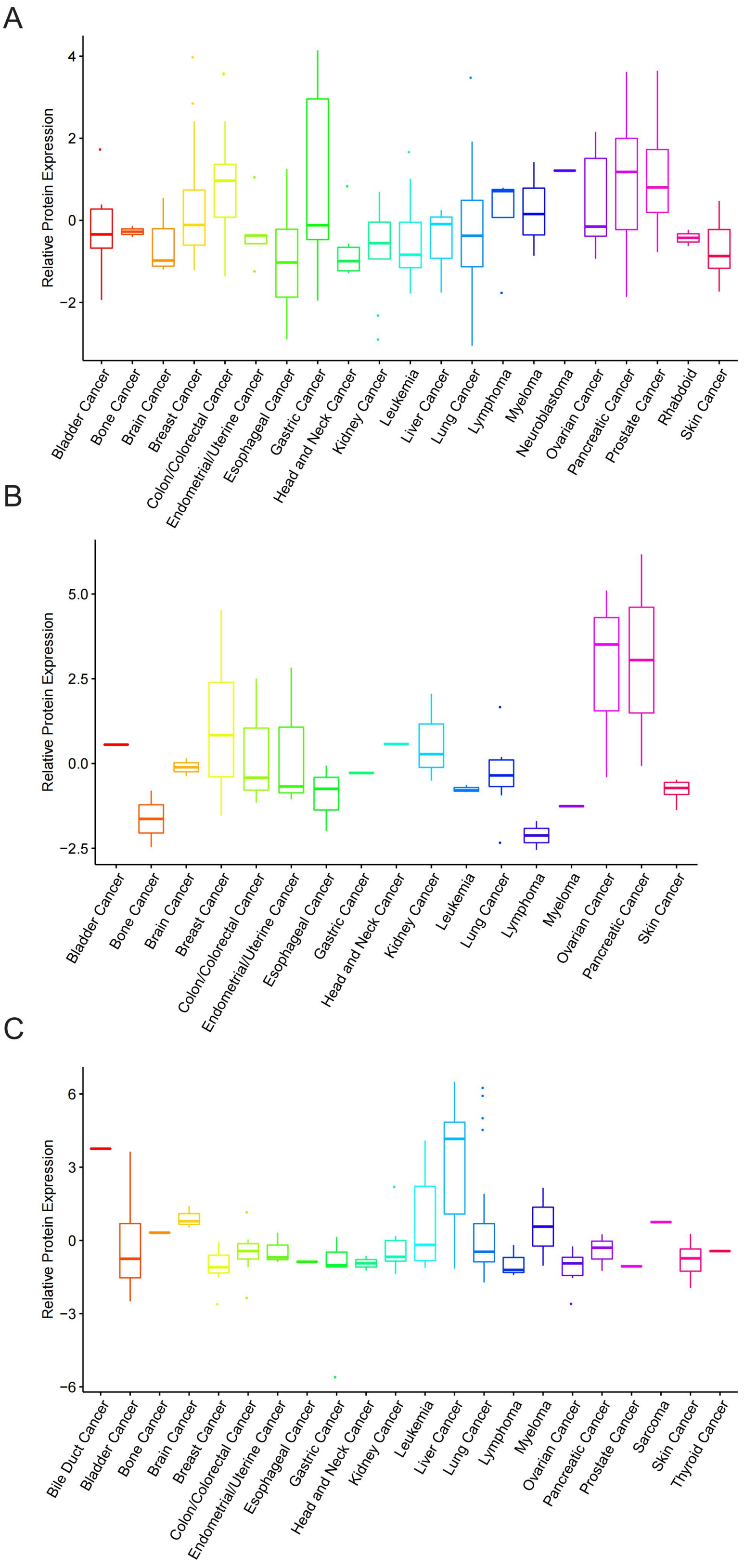

Supplementary Figure 1. Relative protein expression of (A) ACE, (B) ACE2 and (C) AGT in variant types of tumor from Dapmap proteomics data.

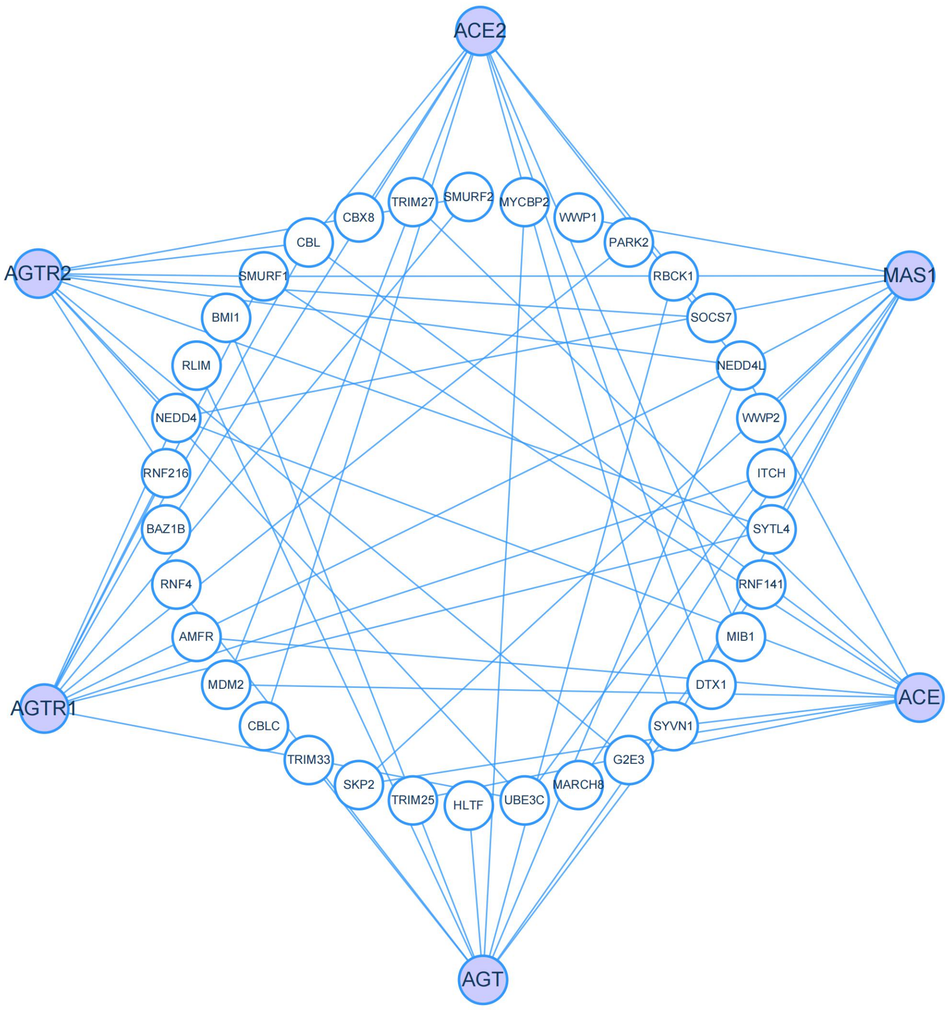

Supplementary Figure 2. Interaction network of ubiquitin ligase (E3 ligase) substrate and individual RAS members from UbiBrowser data.

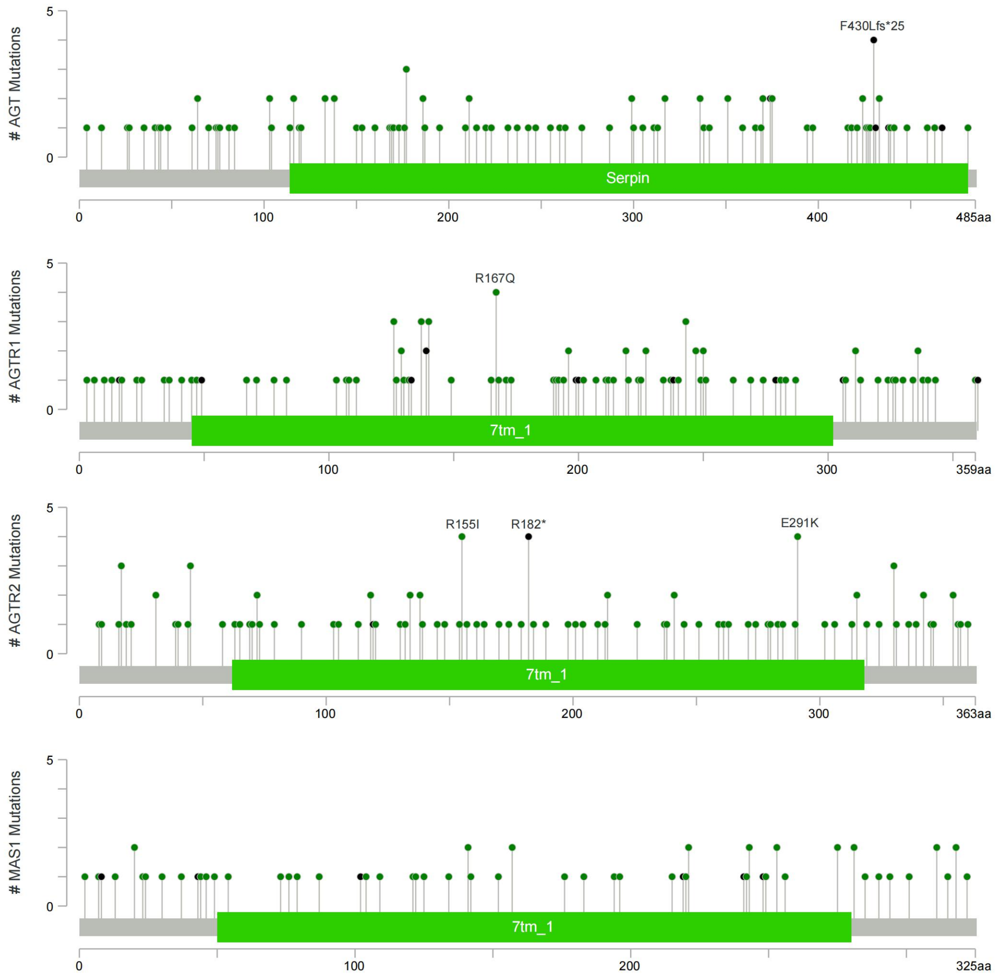

Supplementary Figure 3. Oncoprint for genetic alterations of AGT, AGTR1, AGTR2, MAS1 in TCGA from cbioportal.

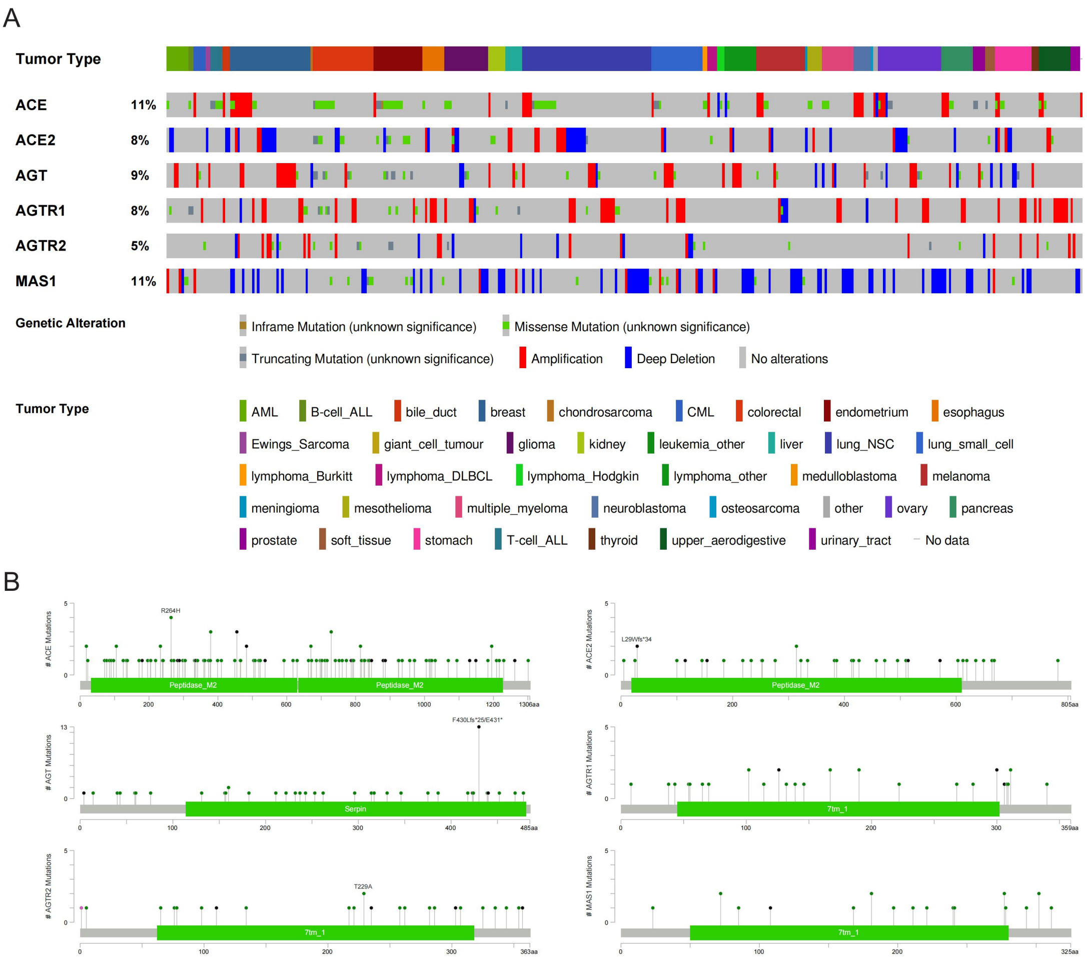

Supplementary Figure 4. Genomic alteration of RAS in CCLE database. (A) Oncoprint for genetic alterations of RAS in CCLE from cbioportal. Each vertical bar points to each sample. (B) Hotspot for amino acid mutation of members of RAS in CCLE.

DATASET	CANCER TYPE	ENDPOINT	N	HR [95% CI]	P−VALUE
**ACE**					
GSE4475	B−cell lymphoma	OS	158	3.60 [1.28 − 10.15]	0.015
E−TABM−346	DLBCL	EFS	53	1.85 [1.02 − 3.35]	0.043
GSE2034	Breast cancer	DMFS	286	0.75 [0.58 − 0.96]	0.025
GSE17537	Colorectal cancer	DSS	49	0.03 [0.00 − 0.56]	0.019
DUKE−OC	Ovarian cancer	OS	133	4.55 [1.35 − 15.31]	0.014
GSE26712	Ovarian cancer	DFS	185	0.39 [0.19 − 0.84]	0.016
GSE26712	Ovarian cancer	OS	185	0.34 [0.15 − 0.77]	0.010
**ACE2**					
GSE4412	Glioma	OS	74	0.44 [0.26 − 0.77]	0.004
GSE12276	Breast cancer	RFS	204	1.12 [1.02 − 1.22]	0.016
GSE6532	Breast cancer	RFS	87	3.36 [1.21 − 9.30]	0.020
GSE6532	Breast cancer	DMFS	87	3.36 [1.21 − 9.30]	0.020
GSE1379	Breast cancer	RFS	60	1.36 [1.08 − 1.73]	0.010
GSE2990	Breast cancer	DMFS	125	1.55 [1.02 − 2.34]	0.038
GSE7390	Breast cancer	DMFS	198	1.19 [1.04 − 1.36]	0.014
GSE7390	Breast cancer	OS	198	1.23 [1.08 − 1.41]	0.003
GSE22138	Uveal melanoma	DMFS	63	0.00 [0.00 − 0.52]	0.034
GSE4573	Lung squamous cell carcinoma	OS	129	0.70 [0.54 − 0.92]	0.009
GSE9891	Ovarian cancer	OS	278	0.63 [0.40 − 1.00]	0.049
E−DKFZ−1	Renal cell carcinoma	OS	59	0.17 [0.04 − 0.77]	0.021
**AGT**					
GSE6532	Breast cancer	RFS	87	0.70 [0.50 − 0.98]	0.036
GSE6532	Breast cancer	DMFS	87	0.70 [0.50 − 0.98]	0.036
GSE17536	Colorectal cancer	DFS	145	0.35 [0.19 − 0.63]	0.001
GSE17536	Colorectal cancer	DSS	177	0.61 [0.38 − 0.99]	0.047
GSE14333	Colorectal cancer	DFS	226	0.61 [0.43 − 0.86]	0.005
GSE31210	Lung adenocarcinoma	OS	204	1.66 [1.09 − 2.52]	0.018
DUKE−OC	Ovarian cancer	OS	133	1.56 [1.14 − 2.14]	0.005
GSE26712	Ovarian cancer	OS	185	1.34 [1.03 − 1.76]	0.031
GSE26712	Ovarian cancer	DFS	185	1.35 [1.05 − 1.73]	0.021
GSE19234	Melanoma	OS	38	2.21 [1.07 − 4.55]	0.032

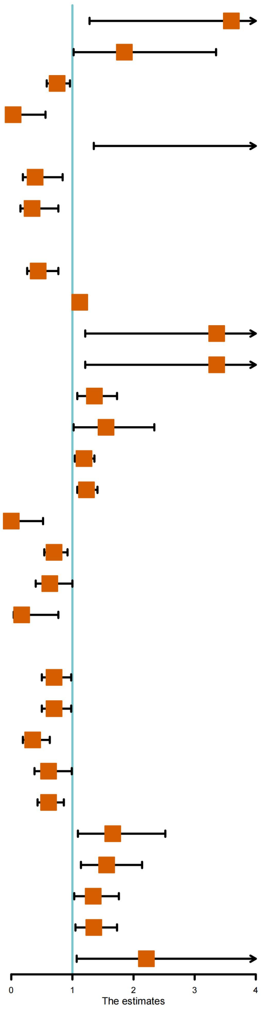

Supplementary Figure 5. Forest-plot for meta survival analysis of ACE, ACE2, and AGT from PrognoScan.

DATASET	CANCER TYPE	ENDPOINT	N	HR [95% CI]	P−VALUE
**AGTR1**					
GSE5122	AML	OS	58	1.51 [1.07 − 2.12]	0.020
GSE12276	Breast cancer	RFS	204	0.84 [0.76 − 0.93]	0.001
GSE1378	Breast cancer	RFS	60	0.89 [0.80 − 1.00]	0.047
GSE1456	Breast cancer	DSS	159	0.40 [0.26 − 0.61]	0.000
GSE1456	Breast cancer	RFS	159	0.52 [0.36 − 0.75]	0.000
GSE3494	Breast cancer	DSS	236	0.67 [0.50 − 0.90]	0.007
GSE14333	Colorectal cancer	DFS	226	1.36 [1.01 − 1.83]	0.044
GSE17537	Colorectal cancer	DFS	55	1.65 [1.03 − 2.66]	0.038
GSE13213	Lung adenocarcinoma	OS	117	0.60 [0.38 − 0.95]	0.031
GSE31210	Lung adenocarcinoma	OS	204	0.36 [0.21 − 0.61]	0.000
GSE31210	Lung adenocarcinoma	RFS	204	0.32 [0.22 − 0.47]	0.000
GSE9891	Ovarian cancer	OS	278	2.73 [1.56 − 4.78]	0.000
GSE26712	Ovarian cancer	DFS	185	1.44 [1.10 − 1.90]	0.009
GSE26712	Ovarian cancer	OS	185	1.48 [1.11 − 1.96]	0.007
GSE30929	Liposarcoma	DRFS	140	0.42 [0.27 − 0.66]	0.000
**AGTR2**					
GSE12417	AML	OS	79	3.20 [1.12 − 9.14]	0.030
GSE8970	AML	OS	34	0.59 [0.38 − 0.92]	0.019
GSE16131	Follicular lymphoma	OS	180	1.21 [1.00 − 1.47]	0.046
GSE16581	Meningioma	OS	67	0.00 [0.00 − 0.82]	0.044
GSE14333	Colorectal cancer	DFS	226	1.40 [1.04 − 1.88]	0.026
GSE22138	Uveal melanoma	DMFS	63	0.00 [0.00 − 0.24]	0.039
GSE31210	Lung adenocarcinoma	RFS	204	0.74 [0.56 − 0.98]	0.038
**MAS1**					
GSE16131	Follicular lymphoma	OS	180	0.74 [0.57 − 0.97]	0.030
GSE7696	Glioblastoma	OS	70	0.25 [0.06 − 0.99]	0.048
GSE4412	Glioma	OS	74	0.58 [0.39 − 0.88]	0.010
GSE19615	Breast cancer	DMFS	115	8.03 [1.07 − 60.05]	0.042
GSE2034	Breast cancer	DMFS	286	0.71 [0.52 − 0.98]	0.038
GSE17537	Colorectal cancer	DFS	55	0.06 [0.01 − 0.65]	0.020
GSE22138	Uveal melanoma	DMFS	63	0.00 [0.00 − 0.00]	0.005
GSE30929	Liposarcoma	DRFS	140	0.06 [0.01 − 0.30]	0.001

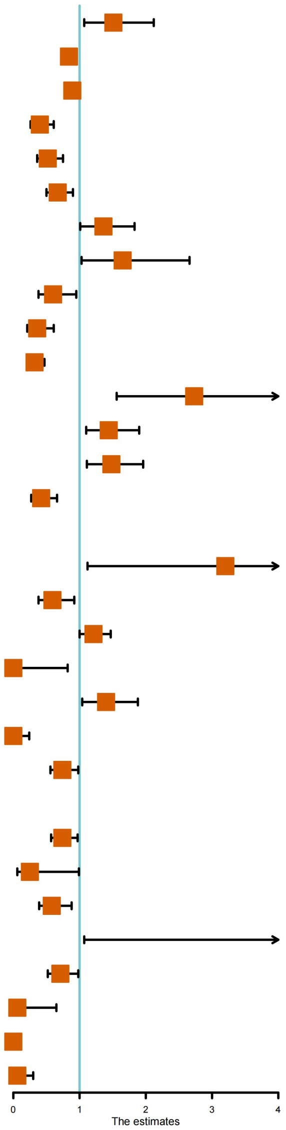

Supplementary Figure 6. Forest-plot for meta survival analysis of AGTR1, AGTR2, and MAS1 from PrognoScan.

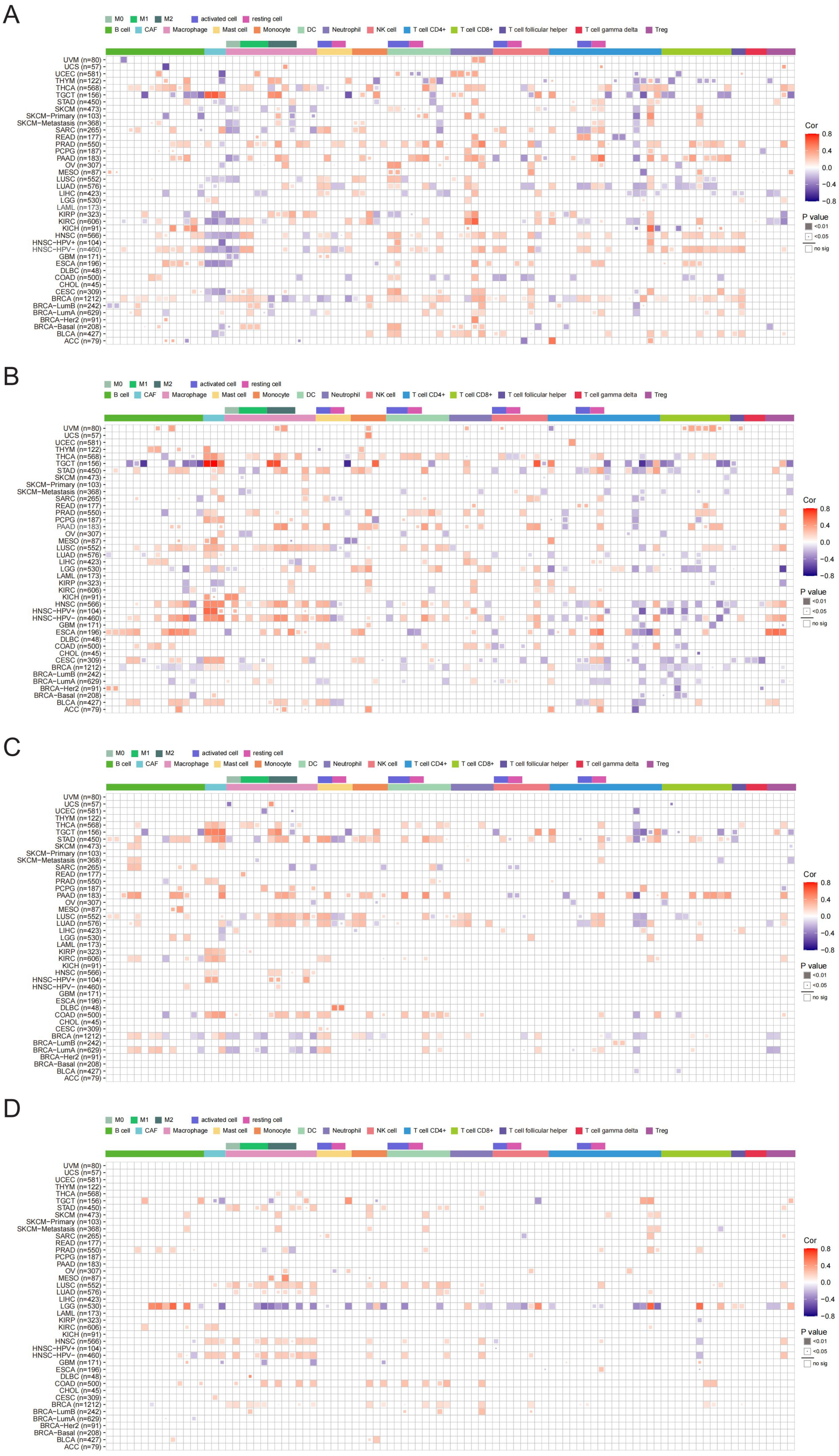

Supplementary Figure 7. Heatmap for correlation between immune cell infiltration and mRNA expression of (A) ACE2, (B) AGT, (C) AGTR2 and (D) MAS1 integrated with multiple databases including CIBERSORT, TIMER, XCELL, EPIC, MCQUANTER, QUANTISIQ. All results were adjusted by tumor purity.

Supplementary table 1

Resources	Url	PMID
UCSC Xena	https://xena.ucsc.edu/	32444850
CPTAC	https://cptac-data-portal.georgetown.edu/	24972168
PrognoScan	http://dna00.bio.kyutech.ac.jp/PrognoScan/index.html	19393097
UbiBrowser	http://ubibrowser.ncpsb.org/2746	28839186
RegNetwork	http://www.regnetworkweb.org/	26424082
LncRNA2Target2.0	http://123.59.132.21/lncrna2target/	30380072
cBioportal	http://www.cbioportal.org/	22588877
TCGA Fusion Gene Database	https://www.tumorfusions.org/	29099951
GSCALite	http://bioinfo.life.hust.edu.cn/web/GSCALite/	29790900
m6A2Target	http://m6a2target.canceromics.org	32392583
TIMER2.0	http://timer.cistrome.org/	32442275
GeneMANIA	http://genemania.org	20576703
